# Ligands binding diffusively to protein target act as inhibitors of protein-protein interactions

**DOI:** 10.1371/journal.pcbi.1013495

**Published:** 2025-09-17

**Authors:** William Jeffries, Bryan M. Delfing, Xavier E. Laracuente, Xingyu Luo, Audrey Olson, Kenneth W. Foreman, Kyung Hyeon Lee, Greg Petruncio, Vito De Benedictis, Mikell Paige, Kylene Kehn-Hall, Christopher Lockhart, Dmitri K. Klimov

**Affiliations:** 1 School of Systems Biology, George Mason University, Manassas, Virginia, United States of America; 2 Department of Chemistry and Biochemistry, George Mason University, Manassas, Virginia, United States of America; 3 Center for Molecular Engineering, George Mason University, Manassas, Virginia, United States of America; 4 Department of Biomedical Sciences and Pathobiology, Virginia-Maryland College of Veterinary Medicine, Virginia Polytechnic Institute and State University, Blacksburg, Virginia, United States of America; 5 Center for Emerging, Zoonotic, and Arthropod-Borne Pathogens, Virginia Polytechnic Institute and State University, Blacksburg, Virginia, United States of America; University of Massachusetts Amherst, UNITED STATES OF AMERICA

## Abstract

Nuclear localization signal (NLS) sequence from capsid protein of Venezuelan equine encephalitis virus (VEEV) binds to importin-*α* transport protein and clogs nuclear import. Prevention of viral NLS binding to importin-*α* may represent a viable therapeutic route. Here, we investigate the molecular mechanism by which two diffusively binding inhibitors, DP9 and DP9o, interfere with the binding of VEEV’s NLS peptide to importin-*α*. Our study uses all-atom replica exchange molecular dynamics simulations, which probe the competitive binding of the VEEV NLS fragment, the coreNLS peptide, and the inhibitors to importin-*α*. Our previous simulations of non-competitive binding of the coreNLS, in which it natively binds to importin-*α*, are used as a reference. Both inhibitors abrogate native peptide binding and reduce the fraction of its native interactions, but they fail to prevent its non-native binding to importin-*α*. As a result, these inhibitors turn the coreNLS into diffusive binder, which adopts a manifold of non-native binding poses. Competition from the inhibitors compromises the free energy of coreNLS binding to importin-*α* showing that they reduce its binding affinity. The inhibition mechanism is based on masking the native binding interactions formed by the coreNLS amino acids. Surprisingly, ligand interference with the binding interactions formed by importin-*α* amino acids contributes little to inhibition. We show that DP9 is a stronger inhibitor than DP9o. By comparative analysis of DP9 and DP9o interactions we determine the atomistic reason for a relative “success” of DP9, which is due to the intercalation of this inhibitor between the side chains of NLS lysine residues. To test our simulations, we performed AlphaScreen experiments measuring IC50 values for the inhibitors. AlphaScreen data confirmed *in silico* ranking of the inhibitors. By combining our recent studies, we discuss the putative mechanism by which diffusively binding inhibitors impact protein-protein interactions.

## Author summary

Although Venezuelan equine encephalitis virus (VEEV) is a pathogen with the potential for epidemic outbreaks, no FDA-approved VEEV antivirals exist. Nuclear localization signal (NLS) sequence from VEEV capsid protein binds to importin-*α* transport protein and clogs nuclear import. Consequently, prevention of viral NLS binding to importin-*α* may represent a viable therapeutic route. We investigate the molecular mechanism by which two inhibitors, DP9 and DP9o, interfere with the binding of VEEV’s NLS peptide to importin-*α*. The unusual property of these inhibitors is diffusive binding to importin-*α*. We used all-atom replica exchange molecular dynamics simulations to probe competitive binding of the VEEV NLS fragment, the coreNLS peptide, and the inhibitors to importin-*α*. Both inhibitors abrogate native peptide binding turning it into diffusive binder, which adopts a manifold of non-native binding poses. The molecular inhibition mechanism results from masking the native binding interactions formed by the coreNLS, but not importin-*α* amino acids. Inhibition is facilitated by DP9 intercalation between NLS lysine side chains. To test our simulations, we performed AlphaScreen experiments measuring IC50 values of the inhibitors. AlphaScreen data confirmed their *in silico* ranking. We discuss that diffusively binding inhibitors may represent a new class of small molecules hindering protein-protein interactions.

## Introduction

Venezuelan Equine Encephalitis Virus (VEEV) is a positive strand RNA alphavirus infectious to humans and equines [1–3]. The virus is transmitted to humans through mosquito bites and can initially present with flu-like symptoms. These can progress to encephalitis and persistent neurological complications. VEEV has historically been the source of multiple epidemics in the Americas and carries the potential to lead to pandemic. Another potential risk of VEEV is its ability to be employed as a bioweapon. Despite these significant dangers, there is currently no approved vaccine or antiviral to prevent infection or transmission of VEEV [2]. The VEEV viral capsid features a capsid protein (CP) which interferes with the functioning of nuclear pore complex (NPC) [4]. Experimental studies have shown that the CP sequence contains a nuclear localization signal (NLS) capable of binding to importin-a (imp*α*) protein and inhibiting nuclear import [4,5]. The VEEV NLS is classified as monopartite [6], which follows a characteristic motif K(K/R)X(K/R) [7]. Consequently, the extended VEEV NLS region appears as EGPSAKKPKKEA, where the fragment KKPKKE is referred to as coreNLS [8].

It has been suggested that inhibiting the binding between VEEV NLS and imp*α* is a potential approach to VEEV antiviral development [4,9,10]. In our previous studies [11–13], we used molecular dynamics simulations to study binding of two inhibitors from the CL6662 scaffold family (referred to as I1 and I2) and the coreNLS peptide to the major NLS imp*α* binding site. Those studies investigated two binding scenarios, competitive and non-competitive. In the competitive scenario, two molecules, the VEEV coreNLS peptide and an inhibitor, compete for binding to imp*α*, while in the non-competitive scenario only one molecule, a coreNLS or an inhibitor, bind to imp*α*. The non-competitive simulations have shown that I1 and I2 bind to imp*α* diffusively adopting a distribution of poses through the major NLS imp*α* binding site. The non-competitive simulations of the coreNLS have demonstrated that this peptide samples nearly native pose upon binding to imp*α*. The subsequent competitive binding simulations showed that I1 and I2 abrogate native coreNLS binding but do not block its non-native binding to imp*α*. In fact, inhibitor interference forces the coreNLS peptide to adopt a distribution of loosely bound poses. By comparing the free energies of binding of I1 and I2 to the “stand-alone” coreNLS or to imp*α*, we showed that both inhibitors preferentially interact with the latter [14]. This finding argued against I1 and I2 masking the coreNLS sequence before its binding to imp*α*. Binding probabilities and free energy analysis have further indicated that I1 is a more effective inhibitor than I2. Importantly, the *in silico* conclusions were confirmed by AlphaScreen experiments measuring IC50 values for both inhibitors [13].

**Fig 1:**
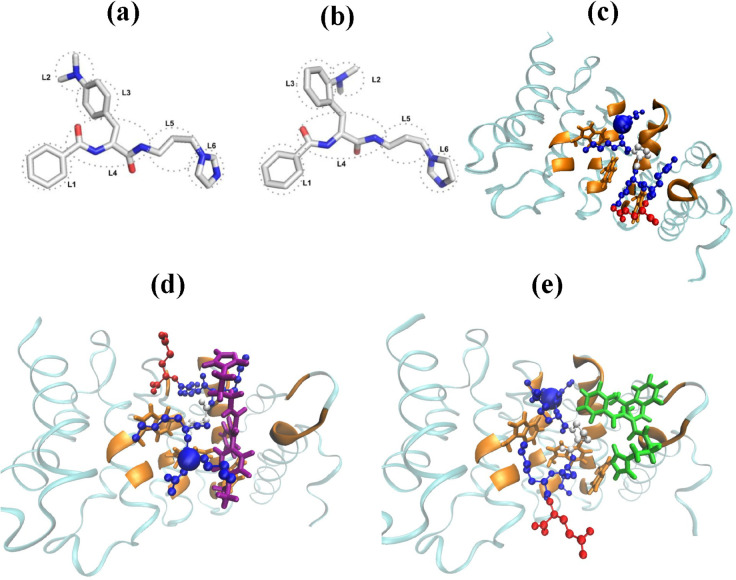
Simulation systems. (A,B) The dominant cluster centroid structures of inhibitors DP9 (a) and DP9o (b) competing with the coreNLS peptide for binding to imp*α*. Structural groups in the inhibitors are indicated. (C) The native pose of the coreNLS peptide bound to imp*α* in 3VE6 PDB structure. In non-competitive binding simulations the coreNLS samples the native-like pose, which primarily deviates from 3VE6 only for the C-terminal residues, Lys10 and Glu11 [12]. (D,E) Representative snapshots of the competitive binding of the coreNLS peptide and DP9 (d) or DP9o (e) inhibitors to imp*α* major NLS binding site. Lysine, proline, and glutamic acid residues in the coreNLS are in blue, beige, and red, whereas DP9 and DP9o molecules are shown in purple and green, respectively. To distinguish the coreNLS and inhibitors in the figure, we used CPK and licorice renderings, respectively. Imp*α* is represented by a blue ribbon, whereas its amino acids natively binding coreNLS peptide are in orange. Spheres mark the Lys6 C*α* atoms in the coreNLS peptides. The figure illustrates that both inhibitors completely abrogate the peptide native pose forcing the coreNLS to bind imp*α* diffusively.

Although the research on I1 and I2 inhibitors suggests that diffusively binding inhibitors can be effective in preventing native binding of NLS sequence to imp*α*, the generality of this conclusion is unclear. Prior structural based design research has identified a lead compound, 1111684 or DP9, as another promising VEEV inhibitor [8]. As evident from Fig 1A this compound has a very different structure that I1 or I2 inhibitors [11] providing an opportunity to compare its inhibition mechanism to I1 and I2. More recently, we engineered a DP9 derivative, referred to as DP9-ortho (DP9o), by moving the dimethyl group to the ortho position (Fig 1B). In this paper, we report the investigation of the inhibiting activities of DP9 and DP9o using all-atom replica exchange molecular dynamics simulations. Specifically, we probe the competitive binding of DP9 or DP9o and the coreNLS peptide to imp*α*. We also investigated the non-competitive binding of these inhibitors to imp*α*. Thus, following the protocol adopted for I1/I2 enables us to compare the activities of these different inhibitors. Our main conclusion is that both DP9 and DP9o, as I1 and I2, bind diffusively to the imp*α* major NLS binding site in non-competitive simulations. In competitive binding simulations, DP9 and DP9o block the coreNLS native binding but did not prevent its overall binding. DP9 showed greater ability to disrupt native peptide binding than DP9o, and the physicochemical rationale behind this finding is discussed. AlphaScreen experiments measuring IC50 values for DP9 and DP9o inhibitors confirmed their *in silico* ranking.

## Methods and models

### Simulation system

Two competitive binding (CB) simulations were conducted, one using the inhibitor DP9 and the other DP9o. A simulation system consisted of an inhibitor molecule (DP9 or DP9o), the coreNLS peptide K_6_KPKKE_11_, a truncated importin-*α* (imp*α*) protein, explicit water molecules, and ions. The reference native structure for imp*α* and bound coreNLS was taken from the 3VE6 PDB entry. Although the imp*α* in 3VE6 is from Mus musculus, it has 98% sequence similarity to the human analogue found in PDB entry 3FEY with the fitted RMSD between the two imp*α* folds being 0.69Å. Only the first 211 residues of the imp*α* from 3VE6 were used to reduce computational cost while retaining the major NLS binding site. The N- and C-termini of imp*α* and coreNLS were capped with acetylated and amidated groups, respectively. A sphere confining the peptide and inhibitor with the radius 18Å was positioned slightly offset from the center of the coreNLS imp*α* binding site. To keep the imp*α* structure close to its 3VE6 fold, while still allowing fluctuations, soft harmonic restraints were applied to C*α* amino acids outside of the sphere. Restraints minimize the deviations of imp*α* without its auto-inhibitory domain from the native fold. The inhibitor DP9 presented in Fig 1A refers to a lead compound [8], which showed an inhibitory effect on VEEV infection. DP9o displayed in Fig 1B is a derivative of DP9, whereby the dimethyl group L2 is moved to the ortho position in the aromatic ring L3.

We used the all-atom CHARMM36m force field [15] to model imp*α* and coreNLS. For DP9 and DP9o, we used the CHARMM General Force field (CGenFF) program version 2.5 with version 4.6 of CGenFF parameters [16,17]. Respectively, 7676 and 7653 water molecules were used to solvate DP9 and DP9o simulations. Water molecules were modeled with the CHARMM-modified TIP3P model [18,19]. To set 150 mM salt concentration and neutralize the overall charge of the system 22 chloride ions and 24 sodium ions were added. The initial unit cell dimensions for the solvated system were approximately 58 Å x 58 Å x 77 Å. The non-competitive binding (NCB) simulation systems had similar design but contained no coreNLS peptide.

### Replica exchange simulations

To sample the interactions between imp*α*, coreNLS, and inhibitor, we used isobaric-isothermal replica exchange with solute tempering (REST) molecular dynamics [20]. A brief outline of REST formalism is given here, with a more comprehensive review provided in the literature [20,21]. In total, the temperatures for *R* = 10 conditions were distributed exponentially from *T*_0_ = 310*K* to *T*_*R*−1_ = 510*K*. Exchanges between replicas *r* and r+1 simulated at temperatures *m* and m+1 occur with the probability $\omega =min[1,e^{-\Delta }]$, where $\Delta =\beta_{m}(H_{m}(X_{r+1})\;-\;H_{m}(X_{r}))\;+\;\beta_{m+1}(H_{m+1}(X_{r})\;-\;H_{m+1}(X_{r+1}))$, $\beta =(RT{)}^{-1}$, *H* is the enthalpy, and *X* represents the system coordinates. Solvent-solvent and solute-solvent interactions at a temperature *T*_*m*_ were scaled by $T_{m}/T_{0}$ and $(T_{m}/T_{0}{)}^{1/2}$ factors, respectively. This scaling excludes solvent-solvent energy contributions from *ω* and reduces the number of replicas without affecting a temperature range and exchange rates. Consequently, the coreNLS peptide and DP9/DP9o ligands were tempered as “hot” solute, the solute-solvent interactions were partially tempered, and the rest of the system (imp*α*, water, and ions) was considered as “cold” solvent. Replica exchanges were attempted at 2 ps intervals, with success rates of approximately 0.34 for all systems. The analysis of technical performance of REST can be found in Supporting Information 1 (S1 Text), including Figs A, B, and C in S1 Text.

Simulations were performed using NAMD [22] with periodic boundary conditions and a 1 fs integration step. Hydrogen-associated covalent bonds were constrained by the SHAKE algorithm. Electrostatic interactions were computed using Ewald summation, and van der Waals interactions were smoothly switched off from 8 to 12 Å. Underdamped Langevin dynamics controlled the temperature with a damping coefficient γ=5ps^−1^. The Nosé-Hoover Langevin piston method set the pressure at 1 atm with piston period and decay of 200 and 100 fs, respectively. The *x*,*y*, and *z* dimensions were coupled.

For each simulation system we have produced four REST trajectories. Their initial structures were prepared as follows. The peptide and an inhibitor (DP9 or DP9o) were placed into the confining sphere. Each system was energy minimized, heated to 310K, and pre-equilibrated at 310K for 2 ns using the NPT ensemble. Then, the systems were further simulated at 700 K for 25 ns using REST energy scaling. This step produced a set of random peptide and inhibitor conformations within the sphere. For each system ten structures, one for each replica, were selected from the 700 K simulations between 16 and 25 ns at an interval of 1 ns. Each selected structure was further equilibrated for 1 ns at the associated REST temperature. Four structures were extracted from each of these 1 ns simulations resulting in each REST replica in each trajectory having a unique initial structure. The DP9 trajectories were 200 ns long. Therefore, the sampling per temperature amounted to 800 ns, and the total sampling at all conditions has reached 8 *μ*s. The convergence analysis is presented in S1 Text, including Figs D, E, and F in S1 Text. Equilibration time in all DP9 trajectories was set at *t*_*eq*_ = 80 ns resulting in 480 ns of equilibrated sampling at 310K. Due to protracted equilibration each DP9o trajectory was simulated for 400 ns, totaling in 1.6 *μ*s per temperature and 16 *μ*s of sampling overall. As shown in S1 Text the 300 ns of sampling at each temperature and each trajectory were discarded as non-equilibrated reducing the equilibrium sampling to 400 ns at 310K. Non-competitive simulations followed similar design with *R* = 5 and equilibration times *t*_*eq*_ being 100 ns for DP9 and 20 ns for DP9o. The total equilibrium sampling at 310K was 400 ns for DP9 and 720 ns for DP9o.

### Computation of structural probes

Binding contacts between imp*α* and coreNLS amino acids or between amino acids and inhibitors occur if any pair of heavy atoms from residues or ligand group is separated by less than 4.5 Å. If any contact is formed between molecules, they are assumed bound. Native contacts between imp*α* and coreNLS were defined using the contacts seen in 3VE6. With this definition the native coreNLS binding site is composed of the following imp*α* amino acids: Leu34, Ser35, Arg36, Glu37, Pro40, Phe68, Trp72, Thr75, Asn76, Ala78, Ser79, Gly80, Thr81, Ser82, Thr85, Gln111, Trp114, Asn118, Asp122 Asn158, and Trp161. These amino acids are displayed in Fig 1C–1E. Using these definitions, we computed *P*_*n*_(*j*) as the fraction of native contacts between coreNLS residue *j* and imp*α* that are retained in the REST simulations. Note that *P*_*n*_(*j*) is normalized by the number of native contacts formed by *j* in 3VE6 structure. Therefore, when, for instance, *j* retains a half of its native contacts, *P*_*n*_(*j*) = 0.5. The fraction *P*_*n*_(*i*) similar to *P*_*n*_(*j*) was defined for imp*α* amino acids *i*. The fraction of non-native contacts *P*_*nn*_(*j*) tracks the formation of binding interactions between the peptide amino acid *j* and imp*α*, which are not found in 3VE6. This quantity is normalized by the total number of contacts between *j* and imp*α* in a sampled structure. Therefore, *P*_*nn*_(*j*) = 0.5 implies that non-native interactions constitute half of all binding interactions formed by *j*. Because of these definitions, in general, $P_{n}(j)\;+\;P_{nn}(j)\neq 1$. We further computed the probabilities, *P*_*b*,*p*_(*i*) and *P*_*b*,*i*_(*i*), which report binding contacts between imp*α* amino acids *i* and coreNLS or inhibitor, respectively. Imp*α* amino acids with top ten such probabilities, either for the peptide or ligand, represented imp*α* binding locations. The probability *P*_*b*,*i*_(*j*) measures binding of inhibitor to the coreNLS amino acid *j*, and *P*_*b*_(*j*,*k*) evaluates the interactions between the coreNLS amino acid *j* and ligand group *k*. For clarity, within the main text we always reserve index *i* for imp*α* amino acids, *j* for coreNLS amino acids, and *k* for ligand groups. Hydrogen bonding was measured using VMD [23] with the donor (D) - acceptor (A) cutoff distance of 3.5 Å and a minimum DHA angle of 135°. The definitions of *π*-cation interactions and tryptophan cage were taken from the previous study [12]. All structural probes are averaged across equilibrated data at *T*_0_ = 310*K*. Standard errors were computed by treating trajectories as individual samples.

### Conformational ensembles and clustering

Clustering of coreNLS peptide poses was performed using the density-based clustering method of Daura *et al* [24]. To prepare the peptides for clustering, the imp*α* structures were first aligned using minimal RMSD between the imp*α* sidechains of the coreNLS binding site. Following protein alignment, the RMSD between all coreNLS conformations were computed. In total, we sampled 10,000 peptide poses from the equilibrated data. Peptide clusters were defined with an RMSD cutoff of *R*_0_ = 2 Å. Only clusters containing at least 1% of the sampled poses were retained. Clustering of ligand binding poses followed the same protocol. Clustering of inhibitor conformations involved their initial alignment and application of the same cutoff.

### Computation of binding free energies

We used the MM-GBSA [25] method for computing the free energy of binding, $\Delta G_{b}$, between coreNLS and imp*α* in the presence of inhibitor. In MM-GBSA, the free energy of a solute is

$$G=E_{mm}+G_{solv,p}+G_{solv,ap}-TS,$$ (1)

where *E*_*mm*_ is the molecular mechanical energy accounting for bonded, electrostatic, and van der Waals interactions, $G_{solv,p}$ is the polar contribution to solvation free energy computed using the Generalized Born implicit solvent model [26], $G_{solv,ap}$ is the apolar contribution to solvation, and *TS* is the solute conformational entropy. For $G_{solv,ap}$ we calculated the apolar solvent accessible surface area with a probe of 1.4 Å radius and set the surface tension coefficient *γ* to 0.005 kcal/mol/Å^2^. The entropy *S* was computed using the Gibbs expression $S=-R_{c}\sum_{E_{mm}}P(E_{mm})lnP(E_{mm})$, where *P*(*E*_*mm*_) is the probability distribution of *E*_*mm*_, for which the bin size of 1 kcal/mol was used. The free energy of binding between coreNLS and imp*α* is

$$\Delta G_{b}=\Delta E_{mm}+\Delta G_{solv,p}+\Delta G_{solv,ap}-T\Delta S,$$ (2)

where $\Delta E_{mm}$, $\Delta G_{solv,p}$, $\Delta G_{solv,ap}$, and TΔS are the changes in the terms from Eq (1) due to binding. The change in the free energy of binding between coreNLS and imp*α* caused by inhibitor *x* (=DP9 or DP9o) is

$$\Delta \Delta G_{b}(x)=\Delta G_{b}(CB;x)-\Delta G_{b}(NCB),$$ (3)

where CB and NCB stand for competitive and non-competitive binding of the peptide to imp*α*, respectively, and the free energy changes are computed using Eq (2) subject to CB or NCB. MM-GBSA method has several limitations, including difficulties in computing entropic contributions and end-point approximation [27,28]. These are further discussed in S1 Text.

Computing $\Delta G_{b}(NCB)$ using MM-GBSA methodology is straightforward. The computation of $\Delta G_{b}(CB;x)$ follows the approach designed in our previous study [13]. We treat inhibitor *x* as an environmental factor, which excludes a part of the coreNLS and imp*α* surface from the interactions with water. Therefore, we substitute them with the inhibitor binding to imp*α* or coreNLS. As a result, the explicit contributions of inhibitor to $\Delta G_{b}(CB;x)$ are omitted. Further, proper computation of $\Delta G_{b}(CB;x)$ requires us to choose if *x* first binds to imp*α* or to the coreNLS before the formation of imp*α*+NLS complex. To distinguish the two scenarios, we used AutoDock Vina [29,30] and compared the binding affinities of *x* to imp*α* and the coreNLS. We found that DP9 and DP9o bind to imp*α* with the respective affinities of −6.9±0.1 and −6.6±0.0 kcal/mol. To probe binding to the coreNLS, we used our previous REST simulations of this peptide in water [13]. We have selected the centroids of the twelve largest peptide clusters, which together represent more than 50% of the 310K conformational ensemble, and used them as binding targets. The average AutoDock Vina binding scores were −4.4±0.0 kcal/mol for DP9 and −4.2±0.1 kcal/mol for DP9o. It follows then that both inhibitors are likely to bind imp*α* prior to the assembly of imp*α*+NLS complex.

## Results

### Competitive binding of the coreNLS peptide and inhibitors to impα

The goal of this research was to study the competitive binding (CB) of the coreNLS peptide KKPKKE and inhibitor, DP9 or DP9o, to imp*α*. The native pose of the coreNLS bound to imp*α* is resolved in the PDB structure 3VE6. Our previous REST simulations of the non-competitive binding (NCB) confirmed that the coreNLS predominantly samples the native pose upon binding to imp*α* [12]. Indeed, 67% of the coreNLS bound poses comprise a native-like cluster with the average RMSD of 2.5±0.1 Å from the native structure. In 3VE6 structure, the coreNLS constitutes a fragment of 12-mer NLS sequence occupying the positions 6-11. Within 3VE6 the coreNLS forms 32 native contacts with the major NLS binding site of imp*α*. Among the key binding interactions are Lys6 forming a salt bridge with imp*α* Asp122 and Lys7 establishing a *π*-cation interaction with Trp161. Furthermore, Lys9 makes its own *π*-cation interactions with Trp72 and Trp114 and resides in a cage formed by these two residues. Lastly, Lys10 forms a salt bridge with Glu37.

As described in Methods and Models we performed preliminary REST simulations probing NCB of DP9 and DP9o inhibitors to imp*α*. Analysis of the DP9 bound ensemble showed that the five top clusters capture from 2 to 4% of the ligand poses together representing merely 13% of the bound ensemble. The investigation of DP9o NCB revealed similar results with top five clusters capturing 11% of poses. In S1 Text we present the imp*α* amino acids with the highest NCB probabilities *P*_*b*,*i*_(*i*) of interacting with both ligands. These top ten imp*α* amino acids listed in Table A in S1 Text were compared with those exhibiting the highest probabilities of binding non-competitively the coreNLS peptide (Table 1) [12]. It follows from this comparison that DP9 and DP9o respectively share six or five such amino acids with the coreNLS. These findings indicate that NCB of both inhibitors to imp*α* is diffusive, i.e., none of them form well-defined binding poses. The analysis of NCB interactions further suggests that both ligands may interfere with the coreNLS binding. Below we explore this possibility.

**Table 1:** Top ten impα amino acids with the highest affinities to bind the coreNLS peptide.^*a*^^,^^*b*^

rank	Non-competing [12]		Competing with DP9		Competing with DP9o	
	amino acid *i*	$P_{b,p}(i)$	amino acid *i*	$P_{b,p}(i)$	amino acid *i*	$P_{b,p}(i)$
1	**Trp161**	0.99±0.00	***Trp161***	0.85±0.00	***Trp161***	0.85±0.08
2	**Ser79**	0.97±0.00	Asp200	0.74±0.00	***Ser79***	0.73±0.12
3	**Asn118**	0.97±0.00	Glu196	0.66±0.01	Asp200	0.72±0.04
4	**Gly80**	0.95±0.00	***Trp114***	0.53±0.00	***Gly80***	0.68±0.15
5	**Ala78**	0.95±0.00	***Ser79***	0.50±0.03	***Asn118***	0.67±0.17
6	**Asp122**	0.95±0.00	***Asn118***	0.38±0.01	***Trp114***	0.64±0.17
7	**Thr85**	0.95±0.00	Arg157	0.37±0.01	Glu196	0.63±0.06
8	**Trp114**	0.92±0.00	***Asp122***	0.36±0.03	***Ala78***	0.62±0.17
9	**Thr81**	0.86±0.00	**Asn158**	0.35±0.02	*Thr85*	0.62 ±0.17
10	**Trp72**	0.86±0.00	***Gly80***	0.35±0.04	***Asp122***	0.62±0.15

*^a^* amino acids in bold belong to the coreNLS native binding site

*^b^* italicized amino acids appear in NCB.

**Fig 2:**
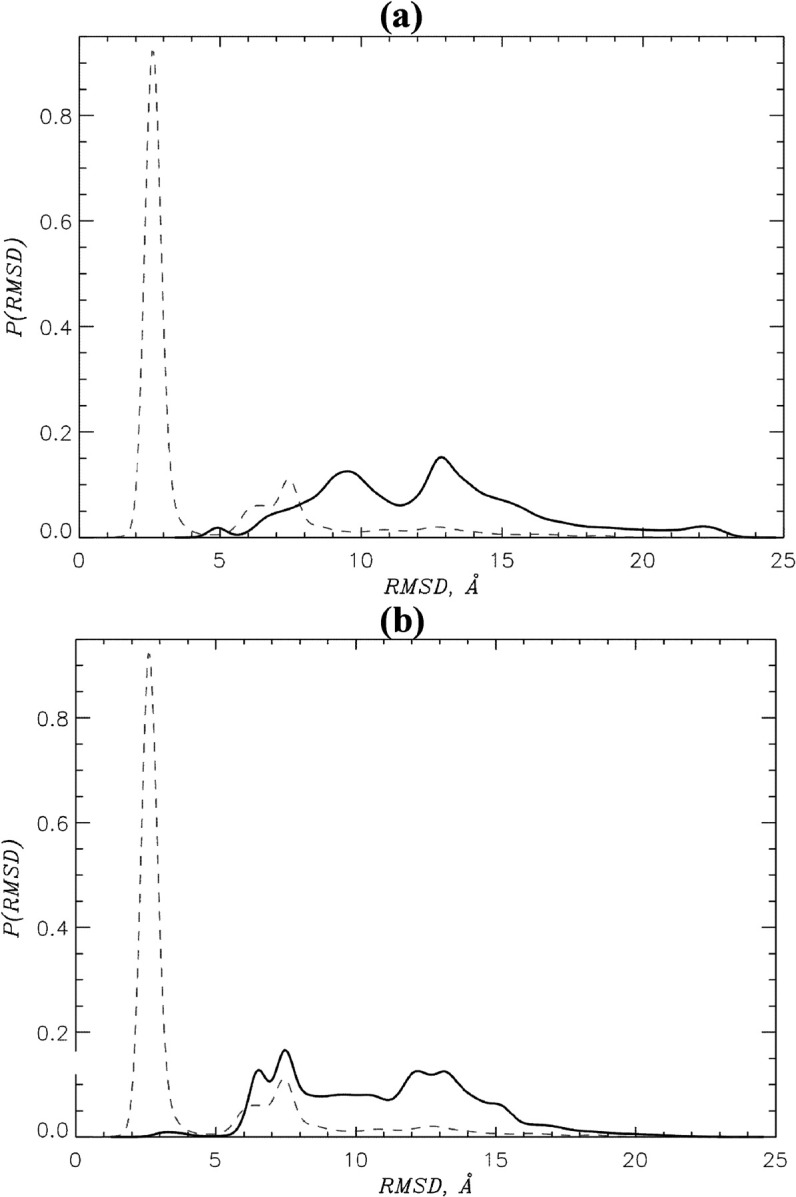
Competitive binding of coreNLS to imp*α.* Probability distributions *P*(*RMSD*) of RMSD values computed between coreNLS binding poses and the native bound pose of the peptide in 3VE6 structure. The coreNLS peptide competes with DP9 (A) or DP9o (B) inhibitors for binding to imp*α*. In both panels continuous and dashed lines correspond to CB and NCB [12], respectively. A multi-peak, broad *P*(*RMSD*) distributions collected in CB simulations implicate diffusive non-native binding of the coreNLS. A narrow unimodal NCB distribution with the peak at ∼2.5 Å shows that in inhibitor-free simulations the peptide binds nearly natively to imp*α*.

To investigate inhibitory activity, we conducted REST simulations of CB of coreNLS peptide and DP9 or DP9o ligand to the major NLS binding site on imp*α*. The results were then compared to those observed without competition from an inhibitor, i.e., upon NCB scenario reported previously [12]. We first computed the overall binding probabilities of coreNLS to imp*α P*_*b*_. We found that for the peptide coincubated with DP9 or DP9o $P_{b}≃1.00\;\pm \;0.00$ and 0.99±0.00, respectively. Since the probability of the coreNLS binding to imp*α* without inhibitor is $P_{b}≃0.97\;\pm \;0.02$, we conclude that the inhibitors do not hinder overall peptide binding. To understand the impact of the inhibitors on the coreNLS binding poses, we computed the probability distributions *P*(*RMSD*) of RMSD values between the CB peptide poses and the 3VE6 native bound structure. Fig 2 compares these distributions with that obtained for NCB [12]. It is evident that both inhibitors dramatically shift *P*(*RMSD*) to the right implicating a non-native binding ensemble. Indeed, while the NCB distribution is unimodal peaking at 2.5Å, the CB *P*(*RMSD*) have multiple peaks at *RMSD*>5 Å. Furthermore, the average ⟨RMSD⟩ for all peptide poses with respect to the 3VE6 structure observed in the presence of DP9 or DP9o are 12.4±0.8 and 10.9±3.8 Å, whereas upon the inhibitor-free binding it is more than two-fold smaller being 4.6±0.4Å. To explore the heterogeneity of peptide binding ensemble, we performed RMSD computations for all-vs-all binding poses and computed resulting clusters. (To obtain all-vs-all RMSD, we computed the fitted RMSD between all pairs of bound structures as described in Methods and Models.) We have shown previously that the fraction of 0.67±0.01 of NCB peptide poses is collected in a single dominant native cluster with the centroid RMSD of 2.5Å from 3VE6 structure [12]. In a sharp contrast, the competition with DP9 reduces the largest cluster population to 0.04 with the centroid RMSD from the native pose of 12.8Å. Upon CB with DP9o the coreNLS forms a dominant cluster containing 27% of all bound poses with the centroid RMSD of 6.8 Å from 3VE6 native pose. For DP9 and DP9o CB simulations the average all-vs-all RMSD of the coreNLS binding poses are 12.4±0.5 and 10.7±4.3 Å.

It is of interest to probe the distributions of inhibitors with respect to the coreNLS and imp*α* observed in CB. We first aligned imp*α* and computed all-vs-all RMSD distributions of inhibitor poses bound to the protein. The average RMSD of DP9 poses was 14.1 Å and their further clustering revealed that the most populated cluster collects 10% of poses. The same computations for DP9o have found the average RMSD of 11.9 Å and the population of the first cluster of 8%. Then, we aligned the coreNLS and recomputed all-vs-all RMSD distributions of inhibitor poses bound to the peptide. The average RMSD of DP9 poses was 16.9Å and the most populated cluster included only 3% of poses. For DP9o we found that the average RMSD is 7.0Å and the first cluster population was 5%. These analyses demonstrate that upon CB both inhibitors bind diffusively to imp*α* and the coreNLS without adopting any specific poses.

To substantiate the findings above, we analyzed the coreNLS binding interactions with imp*α*. Table 2 lists the native and non-native binding probabilities *P*_*n*_(*j*) and *P*_*nn*_(*j*) for each coreNLS residue *j*, as well as the average numbers of binding contacts $\left\langle C_{b}(j)\right\rangle$. Results from NCB and CB simulations are compared. CB reduces $\left\langle C_{b}\right\rangle$ from 28.1 in NCB to 12.7 for DP9 and 16.7 for DP9o, a 55% to 40% reductions, respectively. The overall fraction of retained native contacts, *P*_*n*_(*j*), is reduced from 0.62 in NCB to merely 0.10 for DP9 and 0.23 for DP9o, a striking six- to three-fold drop. Conversely, for both inhibitors the overall fraction of non-native contacts, *P*_*nn*_(*j*), increases more than a factor of two compared to NCB, from 0.29 to 0.74 (DP9) or 0.62 (DP9o). Inspecting the gains and losses caused by DP9 across the individual coreNLS residues *j* we see the largest reduction of native interactions for the first four coreNLS residues. For example, for *j* =  Lys6 *P*_*n*_(*j*) drops from 0.86 upon NCB to 0.14 at CB or, respectively, from 0.65 to 0.03 for Lys9. Simultaneously, the share of non-native interactions formed by Lys9 increases from 0.24 to 0.97. The changes in *P*_*n*_(*j*) and *P*_*nn*_(*j*) caused by DP9o are less drastic. Pro8 features the largest drop in *P*_*n*_(*j*) from 0.75 to 0.23, while its *P*_*nn*_(*j*) rises from 0.11 to 0.55. Notably, DP9 induces the largest decrease in binding affinity at *j* =  Lys6, for which $\left\langle C_{b}(j)\right\rangle$ is reduced by a factor of 3.4. DP9o causes less pronounced loss of binding affinity with that of Lys6, Lys7, or Lys9 being reduced in half. For both inhibitors the fractions of C-terminal native and non-native interactions are respectively low and high in NCB or CB.

**Table 2:** Binding interactions between the coreNLS amino acids and imp*α* protein.

	j= Lys6	j= Lys7	j= Pro8	*j* = Lys9	j= Lys10	j= Glu11	coreNLS peptide
	Non-competing [12]						
*P*_*n*_	0.86±0.01	0.67±0.01	0.75±0.01	0.65±0.00	0.21±0.00	0.36±0.00	0.62±0.02
*P*_*nn*_	0.26±0.00	0.03±0.01	0.11±0.01	0.24±0.01	0.55±0.08	0.75±0.02	0.29±0.02
$\left\langle C_{b}\right\rangle$	10.6±0.1	4.1±0.2	2.6±0.2	5.1±0.2	2.8±0.3	2.9±0.0	28.1±0.4
	Competing with DP9						
*P*_*n*_	0.14±0.03	0.14±0.01	0.18±0.03	0.03±0.01	0.06±0.01	0.09±0.01	0.10±0.02
*P*_*nn*_	0.60±0.07	0.55±0.07	0.68±0.11	0.97±0.04	0.82±0.11	0.89±0.05	0.74±0.09
$\left\langle C_{b}\right\rangle$	3.1±0.2	1.8±0.2	1.6±0.1	1.9±0.2	2.5±0.7	1.6±0.1	12.7±0.8
	Competing with DP9o						
*P*_*n*_	0.41±0.11	0.24±0.07	0.23±0.04	0.18±0.07	0.05±0.02	0.06±0.02	0.23±0.06
*P*_*nn*_	0.29±0.11	0.29±0.01	0.55±0.11	0.64±0.15	0.90±0.03	0.93±0.03	0.62±0.06
$\left\langle C_{b}\right\rangle$	5.2±0.9	2.1±0.4	1.6±0.3	3.0±0.4	3.1±0.6	1.8±0.3	16.7±2.4

We next examined specific binding interactions. First, we partition the number of binding contacts $\left\langle C_{b}\right\rangle$ into those formed with polar and apolar imp*α* amino acids, $\left\langle C_{b,p}\right\rangle$ and $\left\langle C_{b,h}\right\rangle$. Table B in S1 Text shows that DP9 abrogates $\langle \Delta C_{b,p}\rangle =10.0\pm 0.4$ polar and $\langle \Delta C_{b,h}\rangle =5.4\;\pm \;0.5$ apolar contacts. In contrast, for DP9o we find $\langle \Delta C_{b,p}\rangle =5.8\;\pm \;1.7$ and $\langle \Delta C_{b,h}\rangle =5.7\;\pm \;0.7$. Since all but one coreNLS amino acids are charged, DP9 is twice more effective than DP9o in blocking binding electrostatic interactions. Secondly, we considered *π*-cation contacts between the coreNLS Lys residues and imp*α* Trp residues. In NCB, Lys7 forms a stable (with the probability *P* = 0.65) *π*-cation interaction with Trp161, whereas Lys9 establishes such contacts with Trp72 (0.76) and Trp114 (0.56). Strikingly, DP9 eliminates these interactions as their probabilities do not exceed 0.05. Consistent with weaker inhibitory activity of DP9o, this ligand abrogates Lys7 *π*-cation contact, but does so only partially for Lys9, which still forms these interactions with Trp72 and Trp114 with the probabilities of 0.25 and 0.32. Furthermore, in NCB Lys9 resides in the Trp72-Trp114 cage with the probability of 0.58. Both inhibitors completely block occurrences of any Lys in the tryptophan cages.

To inspect the locations of coreNLS binding to imp*α*, we computed the binding probabilities *P*_*b*,*p*_(*i*) for imp*α* residues *i* to form interactions with the peptide. In Table 1, we list the top ten imp*α* residues with highest *P*_*b*,*p*_(*i*) observed in the CB with both inhibitors and compare them against NCB of the peptide. We have shown previously that all top ten amino acids featured in NCB are native, i.e., they form binding interactions with the coreNLS in 3VE6 structure. For these amino acids *P*_*b*,*p*_(*i*) ranges from 0.86 to 0.99. When competing with DP9 or DP9o, the coreNLS peptide binds to ten imp*α* amino acids with the highest *P*_*b*,*p*_(*i*), of which six (DP9) or eight (DP9o) are native and also present in NCB. Furthermore, the number of top ten binding imp*α* amino acids with *P*_*b*,*p*_(*i*) > 0.5 is only five for DP9 or all ten for DP9o. It also of note that apart from Trp161 with the highest *P*_*b*,*p*_(*i*) there is little in common in the ranking of other nine amino acids between DP9 and DP9o.

**Fig 3:**
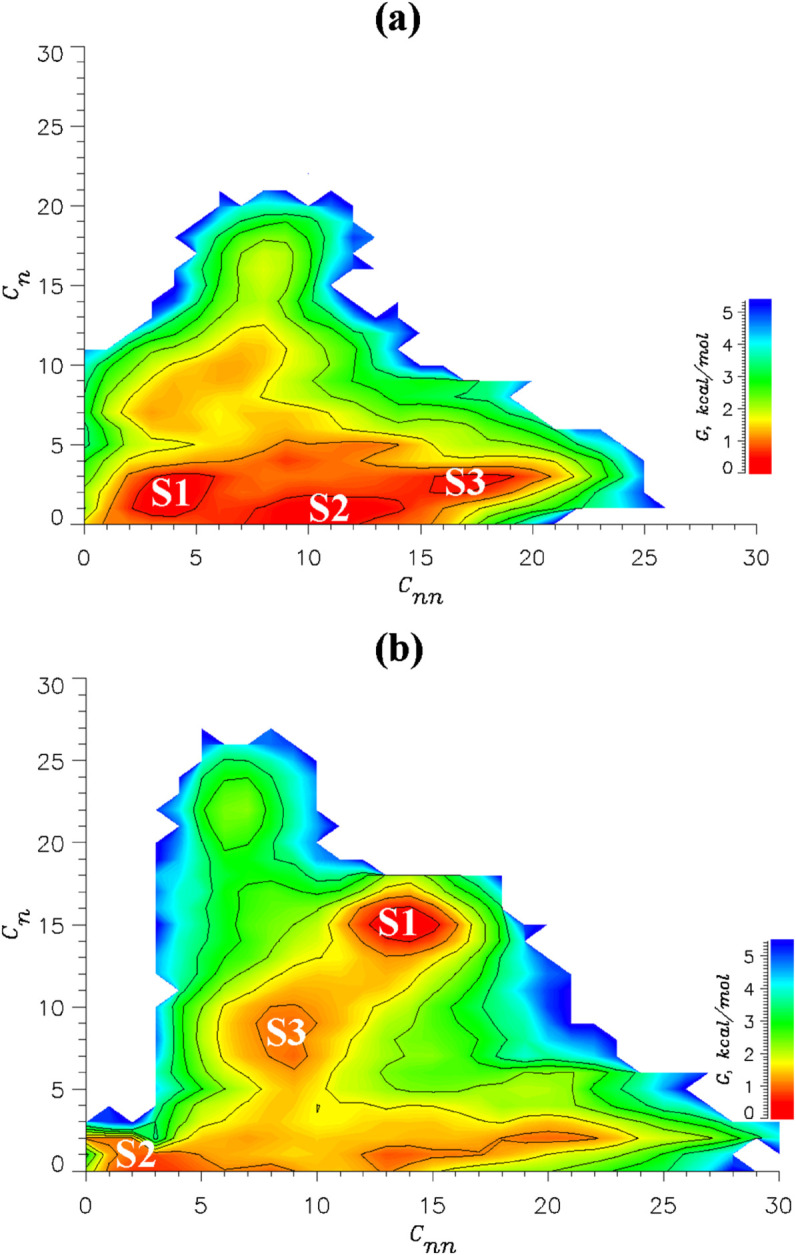
Free energy landscape of coreNLS competitive binding to imp*α.* The two-dimensional binding free energy of coreNLS $G(C_{n},C_{nn})$ is plotted as a function of the numbers of native *C*_*n*_ and non-native *C*_*nn*_ contacts. In panels (A) and (B) the coreNLS peptide competes with the inhibitors DP9 and DP9o, respectively, for binding to imp*α*. The contour lines increments by 0.5 kcal/mol. The low free energy states S1-S3 are marked. In inhibitor-free binding coreNLS populates the state in the upper left corner with $C_{n}∼24$ and $C_{nn}∼5$ [12]. The inhibitors cause a dramatic loss of native interactions as the low free energy state shifts to non-native regions with fewer *C*_*n*_.

To gain additional insight, we computed the coreNLS free energy landscapes observed in CB and compared them against that from NCB [12]. We defined the binding free energy as $G(C_{n},C_{nn})=-RTlnP(C_{n},C_{nn})$, where $P(C_{n},C_{nn})$ is the probability of observing the coreNLS bound state with the numbers of native and non-native contacts with imp*α C*_*n*_ and *C*_*nn*_. The respective free energy landscapes for the coreNLS competing with DP9 and DP9o for binding to imp*α* are shown in Fig 3. In NCB a dominant native free energy basin occurred in the upper left corner with high number of native contacts ($C_{n}∼24$) and minimal number of non-native contacts ($C_{nn}∼5$) [12]. DP9 dramatically shifts this free energy basin to the non-native region composed of states S1-S3 with very few native contacts *C*_*n*_ (∼3) and the number of non-native contacts *C*_*nn*_ ranging from ∼3 to 17. These basins include exclusively non-native poses, from loosely to tightly bound. In contrast, DP9o interference produces a fragmented free energy landscape. It features a purely non-native basin (S2), but also two states S1 and S3, which combine native and non-native interactions. In fact, the dominant bound state S1 is tightly bound with $C_{n}∼C_{nn}∼15$. Notably, the most populated RMSD cluster observed in CB maps exactly onto S1. Comparison of these free energy landscapes with NCB from our previous publication [12] confirms the RMSD analysis that both inhibitors drive the coreNLS peptide away from its native pose, but DP9 is far more effective in blocking native coreNLS binding.

The findings common for DP9 and DP9o can be summarized as follows. First, although both inhibitors do not block the coreNLS from binding to imp*α*, they reduce its binding affinity approximately in half and do not appreciably change the location of peptide binding within the imp*α* major NLS binding site. Second and most importantly, both inhibitors abrogate native peptide binding. DP9 and, to a lesser extent, DP9o transform the coreNLS peptide, which binds natively and specifically to imp*α* major NLS binding site, into diffusive binder, which samples heterogeneous non-native bound ensembles. Third and in line with the previous point, the number of native interactions is reduced from three-fold for DP9o to six-fold for DP9 with concurrent, more than two-fold increase in non-native binding interactions. In NCB most of native binding is localized within the first four coreNLS amino acids, and, consequently, these, particularly Lys6 in case of DP9, undergo the most dramatic loss of native binding. Fourth, both inhibitors largely eliminate *π*-cation binding interactions and wipe out coreNLS Lys side chains caging by imp*α* Trp amino acids. The first and critical distinction between the two inhibitors is potency. DP9 virtually erases the coreNLS native binding leaving no more 10% of it present in CB, whereas DP9o keeps a trace of 20% of native binding interactions. Fig F in S1 Text further illustrates differing inhibiting activities of the ligands by comparing their time dependent RMSD to the native pose. Second, DP9 is far more efficient in interfering with coreNLS electrostatic binding interactions. Indeed, it blocks twice more peptide contacts with imp*α* polar amino acids than DP9o. Thus, DP9 is apparently a more potent inhibitor than DP9o as it produces more heterogeneous and non-native coreNLS binding ensemble.

### Inhibition mechanism

The inhibiting activity of ligands is ultimately measured by the changes in the free energy of forming imp*α*-coreNLS complex. Consequently, we used the MM-GBSA approach [25] described in Methods and Models and computed the changes in the free energy of binding of the coreNLS peptide to imp*α* $\Delta \Delta G_{b}(x)$ caused by the inhibitor *x*. The free energy data are gathered in Tables C, D and E in S1 Text. The major take away from these tables is that interference of DP9 and DP9o increases the binding free energy of the coreNLS peptide by $\Delta \Delta G_{b}($DP9)=24.6 and $\Delta \Delta G_{b}($DP9o)=4.3 kcal/mol, respectively. The analysis of individual contributions to $\Delta \Delta G_{b}(x)$ listed in Table E in S1 Text shows that DP9 reduces the gain in molecular mechanical energy $\Delta E_{mm}$ upon binding by $\Delta \Delta E_{mm}=184.2$ kcal/mol, but it is partially compensated by the decrease in the loss of polar solvation energy $\Delta \Delta G_{solv,p}=-162.5$ kcal/mol. For DP9o we found that $\Delta \Delta E_{mm}=112.2$ kcal/mol and $\Delta \Delta G_{solv,p}=-110.3$ kcal/mol. Several conclusions can be drawn from these data. First, both inhibitors destabilize peptide binding to imp*α* by compromising binding interactions between them as reflected in the reduced gain in $\Delta E_{mm}$. At the same time, inhibitor interference partially offsets the changes in $\Delta E_{mm}$ by limiting the losses in polar solvation. Second, DP9 destabilizes the coreNLS bound state far more dramatically than DP9o. The key factor differentiating the ligands is that the destabilization of coreNLS binding interactions by DP9 outpaces the gain in polar solvation by more than 20 kcal/mol. For DP9o the gap between $\Delta \Delta E_{mm}$ and $\Delta \Delta G_{solv,p}$ contracts to just 2 kcal/mol constituting a 10-fold drop. As explained in S1 Text, specific values of $\Delta \Delta G_{b}$, particularly, a gap of 20 kcal/mol between them for DP9 and DP9o, are likely to be biased due to MM-GBSA limitations but the ranking of inhibitors based on $\Delta \Delta G_{b}$ is expected to be correct (see S1 Text for further details). Thus, these findings are supplemental to our structural analysis of inhibitor interference with the coreNLS binding to imp*α*.

To understand the basis of molecular inhibition, we investigated two hypotheses, which we term “obstruction” and “masking”. A ligand may inhibit the coreNLS binding to imp*α* by “obstructing” the binding interactions formed by imp*α* amino acids and/or by “masking” those formed by the coreNLS. We have already shown above that there is a significant overlap between the imp*α* amino acids binding the coreNLS and inhibitors (Table 1 and Table A in S1 Text) suggesting the possibility of obstruction. To probe its contribution to inhibition, we used CB simulations and computed the correlation between the inhibitor binding probabilities *P*_*b*,*i*_(*i*) to imp*α* amino acids *i* and the changes in imp*α* native binding probabilities caused by inhibitor

$$\Delta P_{n}(i)=P_{n}(i;CB)-P_{n}(i;NCB).$$ (4)

In Eq (4) *P*_*n*_(*i;CB*) and *P*_*n*_(*i;NCB*) refer to the probabilities of native peptide binding by imp*α* amino acid *i* with and without inhibitor competition. If obstruction of imp*α* native binding interactions is responsible for inhibition, this correlation is expected to be high. For DP9 the computed correlation coefficient *r* is -0.12 implying that obstruction does not contribute to inhibition. To confirm, we broaden binding interactions formed by imp*α* to include native and non-native, i.e., we computed

$$\Delta P_{b,p}(i)=P_{b,p}(i;CB)-P_{b,p}(i;NCB),$$ (5)

where *P*_*b*,*p*_(*i;CB*) and *P*_*b*,*p*_(*i;NCB*) are the probabilities of coreNLS binding to *i* with and without inhibitor interference. In this case for DP9 *r* = 0.46 suggesting that obstruction is again not a major factor in inhibition. If we repeat these computations for DP9o, we find that the respective two correlation coefficients are -0.04 and 0.01. Thus, neither DP9 nor DP9o contribute to obstruction.

With obstruction eliminated as a principal basis for inhibition, we considered a masking hypothesis, whereby inhibitor binding to coreNLS masks its binding interactions with imp*α*. To this end, we computed the correlation between the probabilities *P*_*b*,*i*_(*j*) of inhibitor binding to coreNLS amino acid *j* and the loss of native binding interactions formed by *j*,

$$\Delta P_{n}(j)=P_{n}(j;CB)-P_{n}(j;NCB).$$ (6)

In Eq (6) *P*_*n*_(*j;CB*) and *P*_*n*_(*j;NCB*) stand for the fractions of native binding of coreNLS amino acid *j* to imp*α* with and without inhibitor interference. For DP9 this correlation is exceptionally strong with *r* = −0.95. If we relax the definition of $\Delta P_{n}(j)$ to include all, native and non-native, binding interactions, then *r* = −0.55. If we repeat these computations for DP9o, then the respective correlation coefficients are -0.78 and -0.32. This analysis points to masking of coreNLS amino acids as the key factor in inhibiting activity of DP9 and DP9o, which targets native binding. In contrast, obstructing imp*α* binding interactions has no impact on blocking native coreNLS binding. We argued in our previous study [13] that this outcome is due to distinct distributions of binding interactions formed by the coreNLS and imp*α* amino acids. Whereas each coreNLS amino acid is responsible for a large share of all binding interactions, a given imp*α* amino acid contributes relatively little to overall binding due to delocalized distribution of binding interactions over multiple imp*α* residues.

### Differences in the inhibition mechanisms of DP9 and DP9o

**Fig 4:**
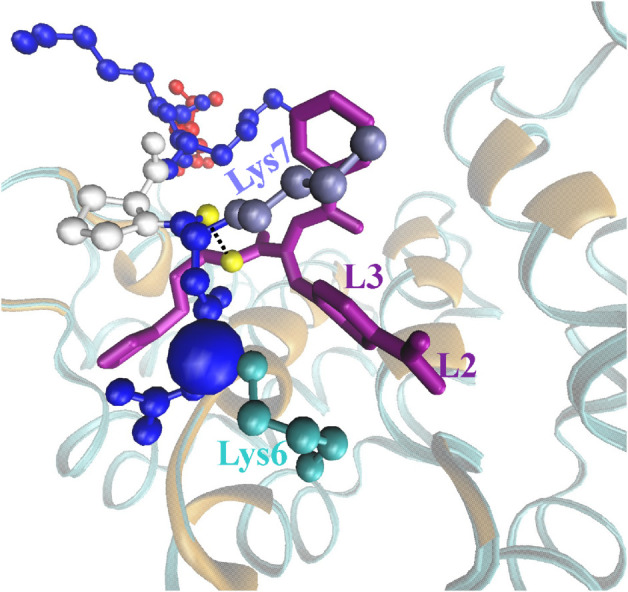
Molecular mechanism of DP9 inhibition. The structural snapshot conveys the molecular mechanism of DP9 inhibition. The molecular representation and coloring follow those used in Fig 1C–1E with the exception of Lys6 and Lys7 shown in cyan and iced blue. A hydrogen bond between the inhibitor nitrogen N21 and the Lys7 backbone oxygen (both in yellow) is shown by dotted line. Inhibitor L3 apolar atoms form hydrophobic contacts with the aliphatic carbons in Lys6 and Lys7 side chains. Supplemented by the hydrogen bonds between DP9 and lysine backbone these interactions contribute to the high inhibition potency of DP9. Repositioning L2 group in DP9o prevents the formation of these interactions resulting in weaker inhibiting activity. Data supporting this inhibition mechanism can be found in Tables I and J in S1 Text.

How does the modification in DP9 chemical structure alter binding of the inhibitor to the coreNLS and imp*α*? We answer this question by first clustering inhibitor conformations in CB. Following Methods and Models we aligned inhibitor structures and computed conformational clusters. Surprisingly, 81% of DP9 structures observed upon CB with the coreNLS can be grouped into a single dominant cluster shown in Fig 1A. Similarly, a DP9o dominant cluster shown in Fig 1B collects 65% of its structures. These computations indicate that despite diffusive binding to imp*α* both inhibitors adopt highly rigid structures. Next, we computed the probabilities *P*_*b*_(*j*,*k*) of contacts between the coreNLS amino acid *j* and inhibitor groups *k*. Tables F, G, and H in S1 Text present *P*_*b*_(*j*,*k*) for DP9 and DP9o. It follows that the strongest interactions (*P*_*b*_(*j*,*k*) > 0.3) between DP9 and the coreNLS involve the groups *k* =  L3 and L4 in the inhibitor and amino acids *j* =  Lys6, Lys7, and Pro8 in the peptide. Additionally, the group L1 exhibits a strong affinity to the coreNLS in general. Strikingly, for DP9o the strongest interactions are shifted to occur between Pro8 and two inhibitor groups L1 and L4. We can quantify the changes in peptide-inhibitor interactions by computing the probability difference $\Delta P_{b}(j,k)=P_{b}(j,k;$DP9o$)\;-\;P_{b}(j,k;$DP9). Then, among all groups L3 reveals the largest loss in binding to the peptide with the interactions of L3 with Lys6 and Lys7 being most compromised ($\Delta P_{b}(j,k)<-0.15$). Interestingly, the overall probability of binding DP9 or DP9o to the coreNLS remains almost unchanged. Furthermore, the difference in DP9 and DP9o chemical structure is solely due to the location of L2, yet its moderate involvement in binding to the peptide is largely unaffected by the modification. More detailed information is provided by Tables I and J in S1 Text, which present the probabilities of contacts between the heavy atoms in L3 group and the side chains of Lys6 and Lys7. It is seen that L3 carbons from DP9 form apolar interactions with aliphatic carbons of both lysines (assuming the probability cutoff of 0.03). In contrast, DP9o loses these apolar contacts. The hydrophobic interactions between L3 and Lys side chains implicate intercalation of the inhibitor ring into the peptide structure as shown in Fig 4 raising the possibility of inhibitor-coreNLS hydrogen bonds. As described in Methods and Models we computed such hydrogen bonds treating inhibitor and peptide polar atoms as potential donors or acceptors. The average number of hydrogen bonds between DP9 and the coreNLS is $\langle N_{hb}\rangle =0.4$, and the most frequent ones (forming with the probability > 0.05) occur between N21 of the inhibitor and the backbone of Lys6 and Lys7. In contrast, for DP9o $\langle N_{hb}\rangle =0.2$, and there are no frequent hydrogen bonds between the inhibitor and the peptide. The extension of this analysis to inhibitor-imp*α* interactions shows that DP9 forms, an average, $\langle N_{hb}\rangle =0.8$ hydrogen bonds, whereas DP9o only 0.4.

Taken together, these results suggest the following atomistic consequences of L2 relocation on inhibiting activity. The interactions between inhibitor and the coreNLS determine the inhibition of the peptide native binding to imp*α*. These interactions are largely due to the hydrophobic contacts between L3 carbons and the side chains of Lys6 and Lys7 (Fig 4). They are supplemented by hydrogen bonds between N21 and the backbones of these amino acids. Relocation of L2 in DP9o to ortho position sterically hinders these interactions eliminating apolar contacts and severely compromising respective hydrogen bonds. As a result DP9o lacks the efficiency of DP9 in masking the native binding interactions formed by Lys6 and Lys7, which represent the “native” anchor in coreNLS binding to imp*α*.

### Experimental assessment of inhibitor activities

**Fig 5:**
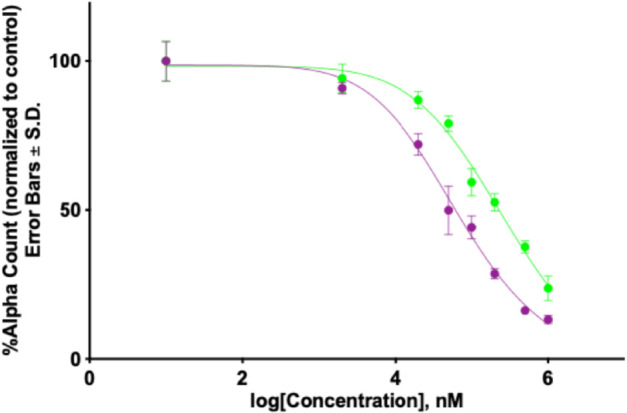
Experimental testing of inhibitor efficacy. Alpha counts for dose-response inhibition assays are plotted. The Y-axis shows the percentage of the alpha counts normalized to the DMSO control. The X-axis presents the inhibitor concentration. Error bars represent the standard deviation and *n* = 6 repeats for each concentration. The IC50 values are reported in *μ*M±SEM. Consistent with the *in silico* binding analysis and free energy computations, the inhibition curves imply that DP9 (in purple) is a stronger inhibitor than DP9o (in green).

To validate inhibiting activities of DP9 and DP9o, we have performed experimental AlphaScreen measurements using the methodology outlined in Supporting Information 2 (S2 Text). The experiments evaluated DP9 and DP9o ligands in terms of their ability to inhibit binding of 12-mer VEEV NLS peptide from 3VE6 structure to imp*α*. The resulting normalized Alpha counts were fitted with the four-parameter nonlinear regression curve $AlphaCount=AlphaCount_{max}\;+\;(AlphaCount_{min}\;-\;AlphaCount_{max})/$ $\left(1\;+\;(IC50/log[inhibitor]{)}^{HillSlope}\right)$. The inhibition plots are shown in Fig 5, from which we extract the IC50 values for DP9 and DP9o as 60±8μM and 232±81μM, respectively. These results are further evaluated in Discussion.

## Discussion

In this study we explored the ability of two ligands, DP9 and DP9o, to inhibit binding of VEEV NLS fragment termed coreNLS to imp*α*. We showed previously that the coreNLS peptide binds natively to imp*α* major NLS binding site forming a pose highly similar to that in 3VE6 structure [12]. The unusual property of these two ligands is their diffusive non-competitive binding to imp*α* presented as a broad distribution of distinct ligand bound poses none of which is dominant. Our results argue that both inhibitors abrogate native peptide binding and reduce the fraction of its native interactions *P*_*n*_ three- to six-fold (Table 3) but they fail to prevent a non-native binding of coreNLS to imp*α*. Competition from DP9 and DP9o increases, i.e., compromises, the free energy of coreNLS binding to imp*α* by $\Delta \Delta G_{b}=24.6$ and 4.3 kcal/mol (Table 3). Thus, inhibitors not only block native coreNLS binding but also reduce its affinity to imp*α*. How common is this class of inhibitors? Previously, we studied two other, structurally distinct, ligands I1 and I2, which also featured a diffusive binding to imp*α* [11,13]. We showed that I1 and I2 also erase the native binding pose of the coreNLS and reduce *P*_*n*_ about four-fold, from 0.62 for NCB to 0.14 and 0.17, respectively, upon CB (Table 3). Importantly, due to I1 and I2 interference the coreNLS binding free energy increases by $\Delta \Delta G_{b}=19.4$ and 8.5 kcal/mol (Table 3). Furthermore, I1 and I2 inhibit coreNLS binding by targeting its native binding interactions, but do not affect those formed by imp*α* amino acids. These observations are similar to those made for DP9 and DP9o above and suggest that despite significant differences in chemical structure, the diffusively binding ligands inhibit coreNLS binding via similar mechanisms, which involve “masking” but not “obstruction”. Thus, the inhibition mechanism reported here may be generic when applied to diffusively binding ligands and small peptides binding to protein targets.

**Table 3:** Summary of *in silico* and experimental activities of diffusively binding inhibitors.

inhibitor *x*	$P_{n}/P_{n}(x)$ ^a^	$\Delta \Delta G_{b}(x)$, kcal/mol	IC50(*x*), μ M
DP9	6.2	24.6	60±8
I1	4.4	19.4	53±6
I2	3.6	8.5	98±30
DP9o	2.7	4.3	232±81

*^a^* $P_{n}/P_{n}(x)$ is the inhibition factor, by which inhibitor *x* reduces the fraction of native contacts *P*_*n*_ retained by the coreNLS in NCB to *P*_*n*_(*x*)eak in CB.

Based on the analysis of retained native interactions and evaluation of coreNLS free energy of binding summarized in Table 3, we rank the inhibitors in terms their efficacy as DP9o < I2 < I1 < DP9. To validate this ranking, we have performed experimental AlphaScreen measurements. The resulting IC50 values for DP9 and DP9o aare 60±8μM and 232±81μM, respectively. The AlphaScreen measurements have previously found that for I1 and I2 IC50 are 53±6μM and 98±30μM [13]. All IC50 values are collected in Table 3 alongside *in silico* findings. Small IC50 are consistent with strong inhibition of NLS binding to imp*α*. Then, the experimental data confirm that the weakest inhibitor is DP9o followed by I2. According to IC50 values, DP9 and I1 offer the strongest inhibition among the four ligands. REST simulations predict DP9 to be slightly better inhibitor than I1, whereas the AlphaScreen data place them within the error margin. Apart from this minor discrepancy, experimental ranking of the inhibitors presented in Table 3 lines up well with *in silico* data. The AlphaScreen data have also the following implication. REST simulations used the 6-mer coreNLS peptide KKPKKE, which is the fragment of 12-mer NLS peptide resolved in 3VE6 structure. Its sequence EGPSAKKPKKEA includes five extra N-terminal and one C-terminal amino acids. However, a good agreement in the experimental and *in silico* rankings of the inhibitors suggests that the mechanisms of inhibiting binding the coreNLS and 12-mer NLS peptide to imp*α* are similar.

Experimental support for ranking the inhibiting activities of the four ligands lends confidence in the inhibition mechanism described *in silico*. Its key component is masking of the native binding interactions formed by the coreNLS. In principle, intermolecular masking of NLS sequences is well documented [31]. For example, masking regulates the nuclear import of the transcription factor NF-κB involved in tumorigenesis, immune and inflammatory responses [32]. Binding of inhibitor protein IκB to NF-κB masks its NLS and subsequently blocks its nuclear import. Phosphorylation of IκB restores accessibility of the NLS making the protein nuclear import ready. Similarly, scaffolding 14-3-3 protein masks the NLS regions in caspase-2 and FOXO forkhead transcription factor proteins preventing their nuclear import [33]. In these scenarios an inhibitor binds to NLS prior to its binding to imp*α*. In our previous study we investigated this possibility for I1 and I2 and found that these inhibitors exhibit much stronger binding affinity to imp*α* than to the coreNLS [14]. That result came from comparing REST binding simulations and from docking analysis. Comparison of DP9 and DP9o docking to imp*α* and the coreNLS peptide also indicates that their free energies of binding to the former are more than 2 kcal/mol lower. As a result, none of these four ligands are likely to mask VEEV NLS prior to its binding to imp*α*, and their primary inhibition mode is a competition with the coreNLS when both are bound to imp*α*. In effect, we propose that imp*α* serves as a template for such binding competition. We argued above that all the four inhibitors “mask” native binding interactions formed by coreNLS amino acids. It is important to clarify that such “masking” occurs when the coreNLS and inhibitor are already bound to imp*α* precluding the possibility of preemptive “masking” prior to binding to imp*α*. It is worth noting that, because coreNLS peptide is disordered in water [14], but adopts a specific pose binding non-competitively to imp*α*, it may follow an “induced fit” binding mechanism [34]. The four inhibitors abrogate the native pose of the coreNLS, but they do not prevent its non-native diffusive binding. We conclude then that the inhibitors target the “induced fit” binding, but not the binding itself.

If the four diffusively binding inhibitors mask the native binding interactions formed by the coreNLS, does it mean that these inhibitors are selective to this NLS? Molecular dynamics studies of SV40 NLS peptide PKKKRKV and the cryptic NLS from the fatty acid binding protein seem to suggest that when bound to imp*α* these peptides undergo significant structural fluctuations [35]. For example, the bound conformational ensemble of SV40 NLS peptide consists of 27 clusters defined with the cut-off of 0.5 Å. The NLS fragment from the critical pluripotency factor Oct4 forms a bound pose in the imp*α* major NLS binding site, which differs from that of the VEEV coreNLS. If so, VEEV coreNLS peptide stands apart from these examples of NLS as it exhibits a nearly native binding pose in the major NLS binding site [12]. We showed earlier that the sequence KKPK occurs in 242 distinct Swiss-Prot human entries, with only five containing the coreNLS sequence KKPKKE [12]. However, only one of these proteins is predicted to bind imp*α* variants. Therefore, due to VEEV NLS rarity the inhibitors may be selective to it, but direct REST competitive simulations with other NLS sequences are needed to evaluate this possibility.

How strongly do our inhibitors destabilize the coreNLS binding to imp*α*? Experimental studies have argued that the free energy of binding of NLS to imp*α* $\Delta G_{b}$ is about -10 kcal/mol [36], although it can be as low as ≈−5 kcal/mol for some human proteins [37]. Recently, we have performed absolute free energy perturbation REST simulations estimating $\Delta G_{b}$ for a shorter KKPK NLS fragment [38]. We found that $\Delta G_{b}=-4.8\;\pm \;0.1$ kcal/mol, and the binding of KKPK is driven by the entropic gains resulting from the partial release of water from lysine solvation shells. The coreNLS differs from KKPK by two additional charged amino acids, lysine and glutamic acid, the latter one showing even better solvation than the former [39]. Furthermore, KKPK binds to imp*α* diffusively similar to the coreNLS coincubated with inhibitors. Then, we can roughly estimate that upon competitive binding the coreNLS $\Delta G_{b}$ is about -8 kcal/mol. If so, the DP9 and I classes of inhibitors compromise the NLS free energy of binding by about 2 kcal/mol. As long as these inhibitors are selective to the VEEV capsid NLS, they are expected to bias nucleus traffic in favor of host proteins. However, even if these diffusively binding inhibitors do not possess selectivity to VEEV, our investigation still showed that they can be effective in abrogating native protein-peptide binding and potentially blocking nuclear import.

## Supporting information

S1 Text Supporting information providing additional simulation details and data. (PDF)

S2 Text Supporting information providing additional experimental details and data. (PDF)
