# Supplementary material for: Ligands binding diffusively to protein target act as inhibitors of protein-protein interactions
Source: PLoS Comput Biol. 2025 Sep 17;21(9):e1013495. doi: 10.1371/journal.pcbi.1013495 (PMC12456801; doi:10.1371/journal.pcbi.1013495)
Supplement: S1 Text — (PDF) [file pcbi.1013495.s001.pdf]

Supporting Information 1

Ligands binding diffusively to protein target act as inhibitors of protein-protein interactions

William Jeffries[1], Bryan M. Delfing[1], Xavier E. Laracuente[1], Xingyu Luo[1], Audrey Olson[1], Kenneth W. Foreman[2], Kyung Hyeon Lee[2,3], Greg Petruncio[2,3], Vito De Benedictis[2,3], Mikell Paige[2,3], Kylene Kehn-Hall[4,5], Christopher Lockhart[1], and Dmitri K. Klimov[1*]

[1]School of Systems Biology, George Mason University, Manassas, Virginia, United States of America

[2]Department of Chemistry and Biochemistry, George Mason University, Manassas, Virginia, United States of America

[3]Center for Molecular Engineering, George Mason University, Manassas, Virginia, United States of America

[4] Department of Biomedical Sciences and Pathobiology, Virginia-Maryland College of Veterinary Medicine, Virginia Polytechnic Institute and State University, Blacksburg, Virginia, United States of America

[5]Center for Emerging, Zoonotic, and Arthropod-borne Pathogens, Virginia Polytechnic Institute and State University, Blacksburg, Virginia, United States of America

*E-mail: dklimov@gmu.edu

REST algorithm performance: Competitive binding (CB) of the coreNLS peptide and the two inhibitors to importin-α (impα) were probed using all-atom replica exchange with solute tempering (REST) molecular dynamics. We analyzed the technical performance of the REST simulations in three ways. First, we plotted the random walks of the replicas across temperatures in the course of the CB simulations (Fig A in S1 Text). For both inhibitors the REST simulations used R=10 conditions with temperatures geometrically distributed from T_0=310K to T_{R-1}=510K (see Models and Methods). If REST sampling behaved as expected, the colored replica walks appear as mosaic.

(a) **(b)**

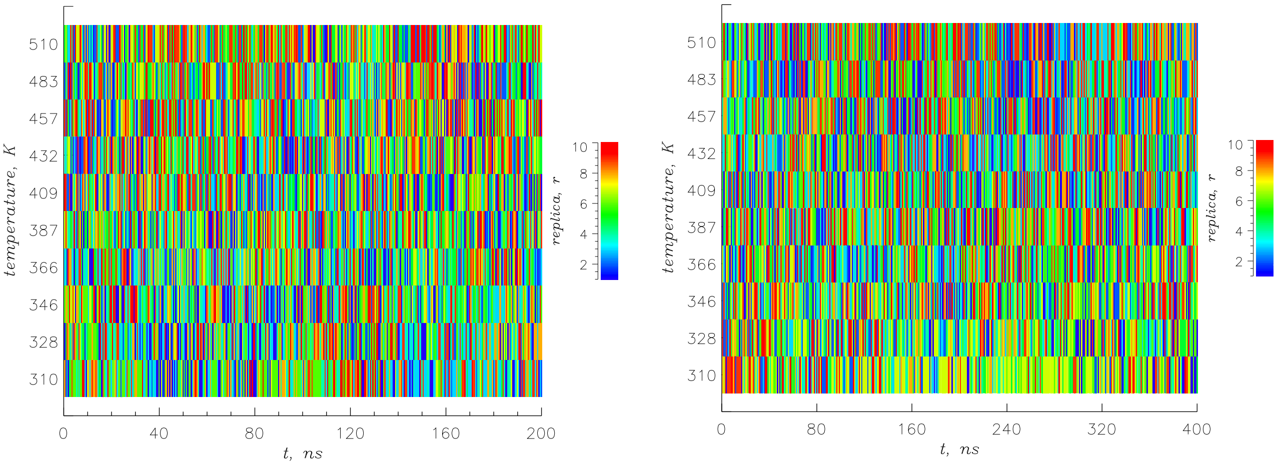

Figure A. Replica walks across ten temperature conditions in one of the trajectories sampling a competition between the coreNLS peptide and DP9 (a) or DP9o (b) inhibitor for binding to impα. The replica color scales represent the initial assignment of replicas to the temperatures.

The second measure of REST sampling performance is the replica mixing parameter [1],

$$m(T) = 1 - \frac{\sqrt{\sum_{r=0}^{R-1} t_r^2}}{\sum_{r=0}^{R-1} t_r}, \tag{1}$$

where T is the REST temperature and t_r is the time spent by replica r at T. The mixing parameter quantifies the distribution of replicas across conditions. The desired theoretical maximum of $m(T)$ indicating randomly mixed replicas is $m_r = 1 - 1/R^{1/2}$. In our case of R=10, the theoretical maximum is 0.68. Fig B in S1 Text displays $m(T)$ across the ten conditions for both CB simulations. It is seen that $m(T)$ for all temperatures approaches m_r. The third method evaluating REST performance involves computing the replica exchange rate $\alpha(T)$ that is the fraction of successful exchanges occurred at temperature T. Optimum ranges for $\alpha(T)$ are 20-40% [2]. Fig C in S1 Text shows that for both inhibitors the exchange rates remain in the acceptable range.

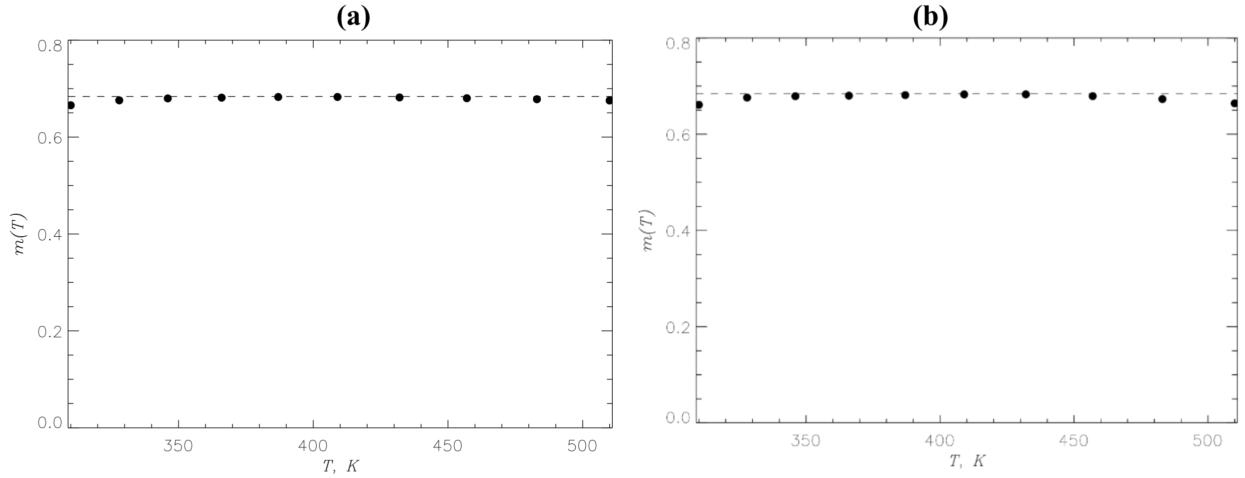

Figure B. The replica mixing parameter $m(T)$ for the CB simulations with DP9 (a) and DP9o (b) inhibitors is plotted as a function of temperature T. The dashed line at m_r=0.68 marks the theoretical maximum of $m(T)$.

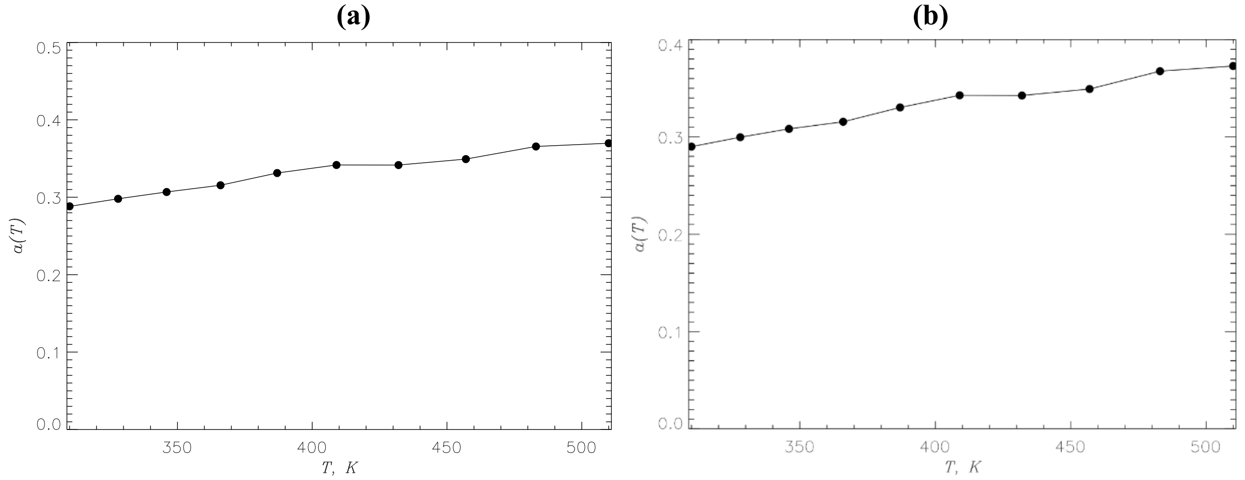

Figure C. Replica exchange rates $a(T)$ are plotted as a function of temperature T for the CB simulations with DP9 (a) and DP9o (b) inhibitors.

Convergence of REST sampling: All binding and conformational analysis was performed for the equilibrated simulation data. Therefore, it is critical to assess the convergence within the equilibrated dataset. CB simulations require monitoring of three different interactions: 1) protein-peptide (impα-coreNLS), 2) peptide-inhibitor (coreNLS-DP9/DP9o), and protein-inhibitor (impα-DP9/DP9o). Fig D in

S1 Text displays these interactions quantified by side chain or inhibitor groups contacts vs REST time *t*. It is seen that all these plots eventually reach their respective baselines.

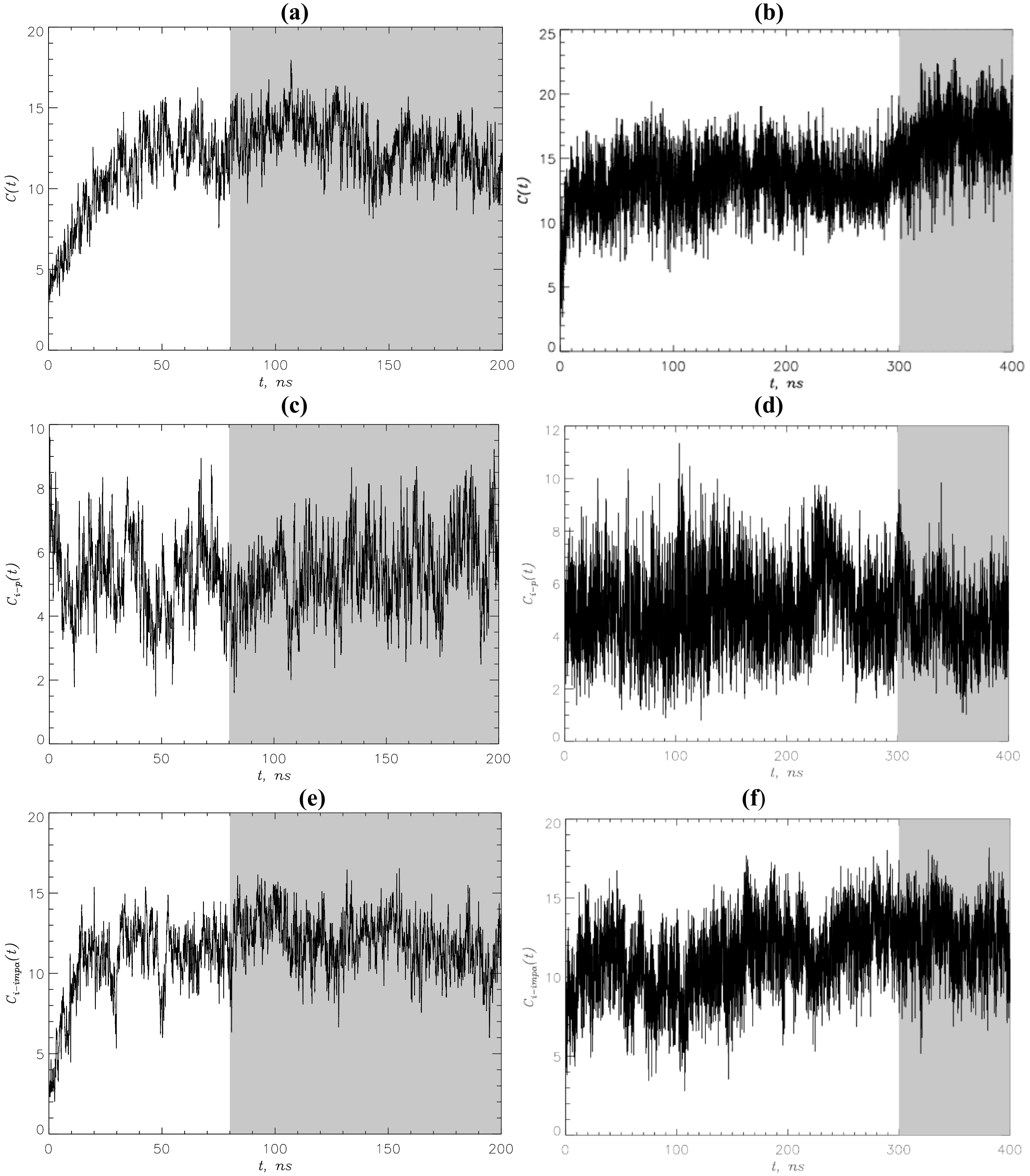

Figure D. (a,b) The total number of binding contacts $C(t)$ between impα and coreNLS amino acids plotted as a function of REST sampling time *t*. (c,d) The total number of contacts $C_{i\text{-}p}(t)$ between coreNLS amino acids and inhibitor groups as a function of REST sampling time *t*. (e,f) The total number of contacts $C_{i\text{-}imp\alpha}(t)$ between impα amino acids and inhibitor groups plotted as a function of REST sampling time *t.* Data in (a,c,e) and (b,d,f) refer to DP9 and DP9o CB, respectively. The data are averaged across four REST trajectories and collected at 310 K. The data are also smoothed with the sliding window of 200 ps. Shaded regions show the equilibrated sampling collected after equilibration times t_{eq}, which are 80 ns for DP9 and 300 ns for DP9o. These times were established using *cRMSD* plots in Fig E in S1 Text.

Analysis of intermolecular contacts in Fig D in S1 Text is one way of determining REST convergence. Additionally, we used a more nuanced methodology for establishing REST convergence based on calculating individual contacts rather than their aggregates as in Fig D in S1 Text. In this method, we first defined a reference contact map $c^{ref}(i,j;n)$ for binding interactions using the initial structure at 310K in trajectory n. In $c^{ref}(i,j;n)$ i and j represent either amino acids or inhibitor groups. Then, instantaneous contact maps $c(i,j;t,n)$ were computed for each time moment t and each trajectory n. The REST timelines were divided into the windows k of 10 ns for DP9 or 20 ns for DP9o. For each window k in a trajectory n we computed the average contact map $C(i,j;k,n)$. Using them we computed the contact root mean squared deviation $cRMSD(k)$

$$cRMSD\ (k) = \left[\frac{1}{N_{tr}}\sum_n \frac{1}{N_c}\sum_{i,j}\left(C(i,j;k,n) - c^{ref}(i,j;n)\right)^2\right]^{\frac{1}{2}}, \qquad (2)$$

where N_{tr} is the number of REST trajectories, N_c is the total number of possible contacts, and k=0, …, 9 for DP9 or 19 for DP9o. Fig E in S1 Text shows $cRMSD(k)$ for impα-coreNLS, coreNLS-DP9/DP9o, and impα-DP9/DP9o interactions. For DP9, impα-coreNLS and impα-DP9 *cRMSD(k)* suggest equilibration after 80 ns, while coreNLS-DP9 *cRMSD(k)* exhibits equilibration after about 40 ns. For DP9o, impα-coreNLS *cRMSD(k)* indicates equilibration after 300 ns, while coreNLS-DP9o and impα-DP9o *cRMSD(k)* reveals either no equilibration or that after about 20 ns. Based on the analysis of Figs D and E in S1 Text, we selected the longest equilibration time t_{eq} among all the quantities for a given inhibitor. Then, t_{eq} is 80 ns for DP9 and 300 ns for DP9o.

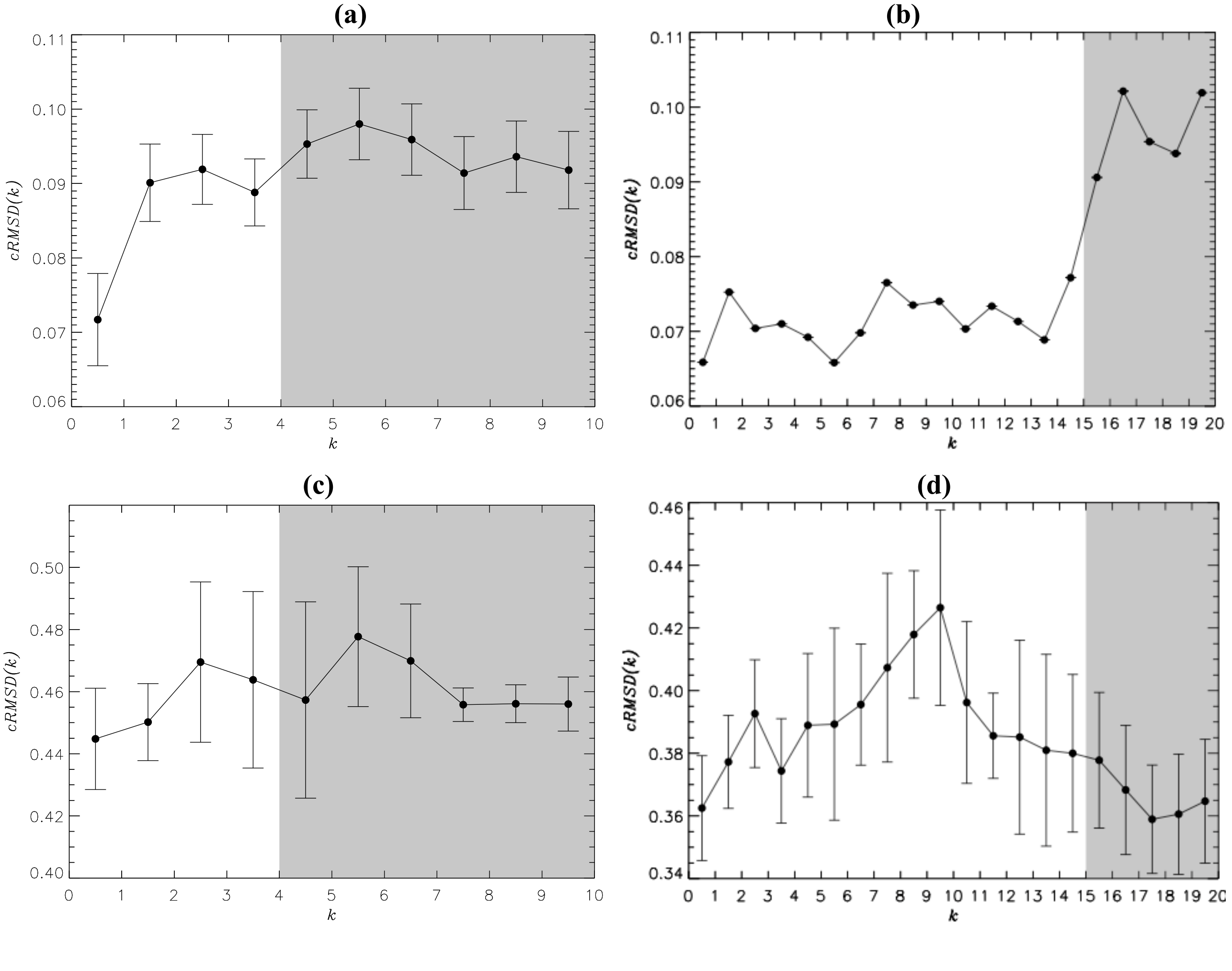

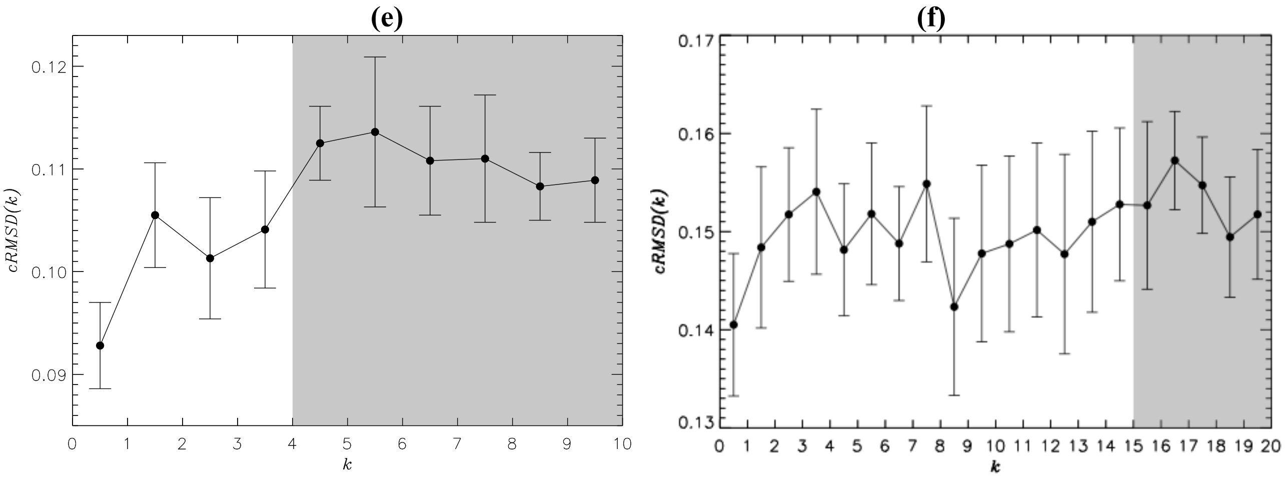

Figure E. The contact root mean squared deviations *cRMSD*(*k*) computed as a function of time windows *k* for various binding interactions. Panels (a) and (b) probe impα-coreNLS contacts in DP9 and DP9o CB simulations, respectively. Panels (c) and (d) show *cRMSD*(*k*) for coreNLS-DP9 and coreNLS-DP9o contacts. Panels (e) and (f) present *cRMSD*(*k*) for impα-DP9 and impα-DP9o contacts. Shaded regions show equilibrated sampling collected after equilibration times t_{eq}, which are 80 ns for DP9 and 300 ns for DP9o CB. The times t_{eq} represent the longest equilibration times among all quantities.

Sampling of native coreNLS binding pose: Initial structures for CB simulations had few native contacts between the coreNLS and impα. Since native pose is abrogated in the CB simulations, it is important to exclude the possibility that this outcome is caused by limited sampling. To map the approach of the peptide to the native binding pose, we plotted the root mean squared deviation (RMSD) between the simulated coreNLS poses at 310K and its native pose in 3VE6 structure. Fig Fa in S1 Text shows the RMSD as a function of REST simulation time *t* for a representative trajectory of DP9o CB. In this trajectory, the coreNLS repeatedly visits poses with < 3 Å RMSD. However, the peptide always escapes these native-like poses to adopt conformations with low native content. The minimum *RMSD* across four DP9o CB trajectories is 1.9 and 1.2 Å for the coreNLS or KKPK fragment of the coreNLS, respectively. KKPK approaches the native-like binding pose within 3 Å with the probability of 0.012. Thus, abrogation of native coreNLS binding is caused by inhibitor rather than inadequate sampling. Fig Fb in S1 Text presents the time dependence of RMSD from the native pose in the representative trajectory of DP9 CB. It is seen that the peptide does not approach the native pose closer than 4.2 Å. In fact, the minimum *RMSD* across all trajectories are still 4.2 for the coreNLS and 2.9 Å for KKPK, respectively. In this context, one should consider the possibility that large RMSD values in DP9 CB are caused by insufficient equilibration. However, three circumstances argue against this assertion. First, we thoroughly tested and confirmed an apparent equilibration of DP9 CB simulations in Figs D and E in S1 Text using three sets of probes. Second, in DP9o CB the coreNLS reaches native-like states (*RMSD* < 3 Å) within the timescale of DP9 CB simulations, i.e., 200 ns. Third, the inhibition mechanism described in the main text explains how DP9 blocks the peptide native binding. Therefore, we conclude that stronger inhibiting propensity of DP9 drives the peptide father away from the native pose.

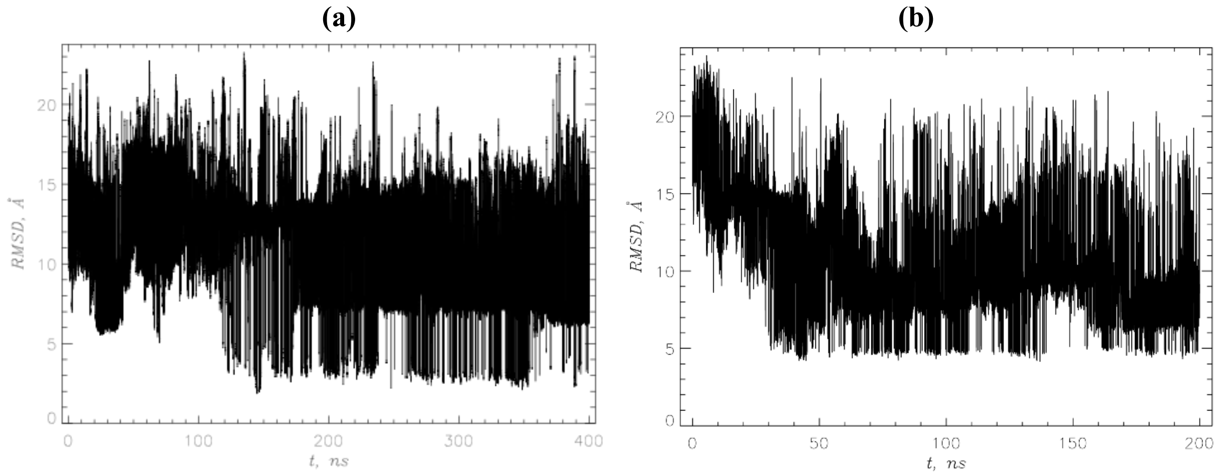

Figure F. The root mean squared deviation *RMSD* of the coreNLS peptide from its native pose as a function of REST time *t*. The native pose corresponds to that in 3VE6 PDB structure. Panels (a) and (b) show the representative trajectories from DP9o and DP9 CB simulations, respectively.

Inhibitor binding to importin-α: We explored the binding of inhibitors DP9 and DP9o to impα, both in CB and NCB simulations. For each simulation, we calculated the probability, $P_{b,i}(i)$, of inhibitor binding to impα amino acid *i*. Table A in S1 Text displays the top ten impα amino acids binding to inhibitors with highest $P_{b,i}(i)$.

Table A Top ten impα amino acids with the strongest affinities toward inhibitors in NCB and CB.[a]

rank	amino acid *i*	$P_{b,i}(i)$	amino acid *i*	$P_{b,i}(i)$	amino acid *i*	$P_{b,i}(i)$	amino acid *i*	$P_{b,i}(i)$
	**Inhibitor DP9**				**Inhibitor DP9o**			
	**Non-competitive binding**		**Competitive binding**		**Non-competitive binding**		**Competitive binding**	
**1**	**Ser79**	0.44±0.02	**Trp161**	0.85 ± 0.00	**Trp114**	0.44±0.04	**Ser79**	0.64±0.07
**2**	**Trp161**	0.35 ± 0.01	Asp200	0.74 ± 0.00	**Ser79**	0.42±0.06	**Asn76**	0.48±0.06
**3**	**Pro40**	0.34 ± 0.01	Glu196	0.66 ± 0.01	**Trp161**	0.40±0.06	**Trp114**	0.47±0.10
**4**	**Trp114**	0.32 ± 0.01	**Trp114**	0.53 ± 0.00	**Asn76**	0.35±0.04	**Gly80**	0.42±0.09
**5**	**Asn76**	0.30 ± 0.01	**Ser79**	0.50 ± 0.03	**Trp72**	0.31±0.05	**Trp161**	0.40±0.08
**6**	Ile42	0.29 ± 0.01	**Asn118**	0.38 ± 0.01	**Asn118**	0.31±0.03	**Pro40**	0.39±0.11
**7**	**Gly80**	0.28 ± 0.01	Arg157	0.37 ± 0.01	Arg168	0.26±0.04	Ile42	0.37±0.10
**8**	Gln39	0.28 ± 0.00	**Asp122**	0.36 ± 0.03	Gly121	0.24±0.04	**Ser35**	0.36±0.10
**9**	**Trp72**	0.26 ± 0.01	**Asn158**	0.35 ± 0.02	**Pro40**	0.24±0.07	**Glu37**	0.34±0.10
**10**	**Asn118**	0.24 ± 0.00	**Gly80**	0.35 ± 0.04	**Thr75**	0.23±0.04	**Asn118**	0.31±0.09

[a] Amino acids in bold belong to native coreNLS binding site

Binding interactions between the coreNLS peptide and impα: We partitioned the number of binding contacts $<C_b>$ between the coreNLS and impα into those formed by polar and apolar impα amino acids, $<C_{b,p}>$ and $<C_{b,h}>$. Table B in S1 Text presents these binding interactions for NCB and CB of the coreNLS with DP9 or DP9o. The data are analyzed in the main text.

Table B The coreNLS-impα binding interactions partitioned between polar and hydrophobic impα amino acids

simulation	$<C_{b,p}>$	$<C_{b,h}>$	$<C_b>=<C_{b,p}>+<C_{b,h}>$
**NCB**	17.7 ± 0.4	10.4 ± 0.1	28.0 ± 0.4
**CB with DP9**	7.7 ± 0.3	5.0 ± 0.5	12.7 ± 0.8
**CB with DP9o**	11.9 ± 1.7	4.7 ± 0.7	16.7 ± 2.4

Binding free energy: We used the MM-GBSA approach (see Model and Methods) to calculate the binding free energy change $\Delta\Delta G_b(x)$ caused by the inhibitor *x*. Table C in S1 Text lists the free energies and their components. The free energy components are defined in Eqs. (1,2) in the main text. For impα-coreNLS-inhibitor systems, both the potential and solvation energies of inhibitor *x* are excluded while the interactions of *x* with impα and/or the coreNLS are included. Table D in S1 Text shows the binding free energy ΔG_b(NCB) computed from NCB simulations [3], the binding free energies ΔG_b(CB; *x*) for CB with the inhibitor *x*, and the binding free energy changes caused by inhibitors $\Delta\Delta G_b(x)$. The contributions to $\Delta\Delta G_b(x)$ are displayed in Table E in S1 Text. These components are the molecular mechanical energy E_{mm}, polar and apolar solvation energies $G_{solv,p}$ and $G_{solv,ap}$, and entropy *TS* listed in Eq. (1).

Table C Free energies and their components for various systems

System	E_{mm}, kcal/mol	$G_{solv,p}$, kcal/mol	$G_{solv,ap}$, kcal/mol	*TS*, kcal/mol	*G*, kcal/mol
**coreNLS peptide**[a]	1492.3	-1697.7	6.2	2.2	-201.1
**Impα**[a]	643.6	-2526.8	54.7	3.1	-1831.5
**Impα+coreNLS**[b]	389.5	-2513.7	54.5	3.3	-2073.0
**Impα-DP9**[c]	594.3	-2503.2	53.4	3.2	-1858.6
**Impα-DP9o**[c]	624.0	-2522.5	54.6	3.2	-1847.1
**Impα+NLS-DP9**[c]	524.3	-2653.6	56.0	3.2	-2075.4
**Impα+NLS-DP9o**[c]	482.1	-2619.6	56.7	3.2	-2084.1

[a] data collected in [4]
[b] data collected in [5]
[c] "minus" in the system name indicates that the contribution from the inhibitor is not counted, while the interaction between the inhibitor and the rest of the complex is included.

Table D Binding free energies and their changes due to inhibitor interference.

**quantity**	**Free energy,** kcal/mol	
ΔG_b**(NCB)**[a]	-40.3	
	**Inhibitor *x*=DP9**	**Inhibitor *x*=DP9o**
ΔG_b**(CB;*x*)**	-15.8	-35.9
$\Delta\Delta G_b(x)$	24.6	4.3

[a] data collected in [4]

Table E Contributions to the changes in the free energy of coreNLS binding to impα

**quantity**	**Inhibitor *x*=DP9**	**Inhibitor *x*=DP9o**
$\Delta\Delta E_{mm}$, kcal/mol	184.2	112.2
$\Delta\Delta G_{solv,p}$, kcal/mol	-162.5	-110.3
$\Delta\Delta G_{solv,ap}$, kcal/mol	2.8	2.3
$\Delta\Delta TS$, kcal/mol	-0.1	-0.1

There are, however, limitations in $\Delta\Delta G_b(x)$ computations [6,7]. The one is related to difficulties in accurate accounting for entropic contributions. The entropic terms present or contributing to Tables C, D, and E in S1 Text may capture the conformational changes in impα, coreNLS, and their complex. However, these entropic terms leave out translational, rotational, and vibrational contributions. The second limitation is related to continuum representation of water, while the third is due to the end-point approximation. The latter limitation arises, because MM-GBSA ignores intermediate bound states. Since it is difficult to ascertain the relative contribution of these factors to DP9 and DP9o competitive binding, the resulting $\Delta\Delta G_b(x)$ must be corroborated with other computational and experimental findings. Because the inhibitors' ranking based on the values of $\Delta\Delta G_b(x)$ is consistent with the analysis of binding interactions and the AlphaScreen experiments, we use MM-GBSA data to describe inhibition energetics.

Analysis of interactions between inhibitors and coreNLS peptide: We computed the probabilities $P_{i,p}(j,k)$ of contacts between the coreNLS amino acid *j* and inhibitor group *k*. Tables F and G in S1 Text present $P_{i,p}(j,k)$ for DP9 and DP9o. Table H in S1 Text contains the probability difference map $\Delta P_{i,p}(j,k) = P_{i,p}(j,k;\text{DP9o}) - P_{i,p}(j,k;\text{DP9})$. The data are analyzed in the main text.

Table F Probabilities $P_{i,p}(j,k)$ of contacts between the coreNLS amino acids *j* and DP9 inhibitor groups *k*.[a]

	Lys6	Lys7	Pro8	Lys9	Lys10	Glu11	All
**L1**	0.22	0.20	0.25	0.15	0.08	0.05	0.50
**L2**	0.10	0.10	0.11	0.10	0.06	0.06	0.34
**L3**	0.32	0.32	0.23	0.16	0.08	0.07	0.53
**L4**	0.31	0.28	0.35	0.22	0.06	0.05	0.58
**L5**	0.17	0.19	0.23	0.13	0.04	0.05	0.40
**L6**	0.15	0.15	0.12	0.14	0.05	0.06	0.37
**All**	0.54	0.53	0.54	0.45	0.23	0.21	0.80

[a] Probabilities $P_{i,p}(j,k)$ in orange represent those exceeding 0.3.

Table G Probabilities $P_{i,p}(j,k)$ of contacts between the coreNLS amino acids *j* and DP9o inhibitor groups *k*.[a]

	Lys6	Lys7	Pro8	Lys9	Lys10	Glu11	All
**L1**	0.28	0.26	**0.33**	0.10	0.07	0.05	0.57
**L2**	0.10	0.11	0.16	0.10	0.04	0.02	0.36
**L3**	0.12	0.15	0.15	0.18	0.14	0.08	0.36
**L4**	0.17	0.17	0.39	0.19	0.11	0.06	0.61
**L5**	0.09	0.09	0.21	0.11	0.06	0.04	0.38
**L6**	0.07	0.06	0.19	0.12	0.08	0.03	0.37
**All**	0.48	0.53	0.65	0.39	0.27	0.15	0.83

[a] Probabilities $P_{i,p}(j,k)$ in orange represent those exceeding 0.3.

Table H Probability difference map $\Delta P_{i,p}(j,k)$ comparing DP9o and DP9 binding to the coreNLS.[a]

	Lys6	Lys7	Pro8	Lys9	Lys10	Glu11	All
L1	0.06	0.06	0.08	-0.05	-0.01	0.00	0.07
L2	0.00	0.01	0.05	0.00	-0.02	-0.04	0.02
L3	-0.20	-0.17	-0.08	0.02	0.06	0.01	-0.17
L4	-0.14	-0.11	0.04	-0.03	0.05	0.01	0.03
L5	-0.08	-0.10	-0.02	-0.02	0.02	-0.01	-0.02
L6	-0.08	-0.09	0.07	-0.02	0.03	-0.03	0.00
Aaa	-0.06	0.00	0.11	-0.06	0.04	-0.06	0.03

[a] Probability differences $\Delta P_{i,p}(j,k)$ in orange represent those below -0.15.

To provide a more intricate analysis of inhibitor-coreNLS interactions, we computed the probabilities of contacts between the heavy atoms in L3 group from the inhibitor and the side chains of Lys6 and Lys7 from the coreNLS peptide (see Fig. 1a,b in the main text). The results are presented in Tables I and J in S1 Text and analyzed in the main text.

Table I Probabilities of contacts between heavy atoms from DP9 L3 group and the side chains of coreNLS Lys6 and Lys7.[a]

	**Lys6**					**Lys7**				
	**CB**	**CG**	**CD**	**CE**	**NZ**	**CB**	**CG**	**CD**	**CE**	**NZ**
**C4**	0.05	0.02	0.02	0.02	0.00	0.00	0.02	0.03	0.05	0.02
**C5**	0.05	0.02	0.03	0.02	0.01	0.01	0.02	0.03	0.03	0.02
**C6**	0.07	0.04	0.03	0.02	0.01	0.02	0.01	0.02	0.02	0.01
**C7**	0.06	0.02	0.02	0.01	0.00	0.01	0.02	0.02	0.02	0.01
**C30**	0.07	0.03	0.02	0.01	0.00	0.02	0.05	0.04	0.03	0.01
**C31**	0.06	0.03	0.02	0.01	0.00	0.01	0.04	0.05	0.05	0.02

[a] Probabilities in orange represent those exceeding 0.03.

Table J Probabilities of contacts between heavy atoms from DP9o L3 group and the side chains of coreNLS Lys6 and Lys7.[a]

	**Lys6**					**Lys7**				
	**CB**	**CG**	**CD**	**CE**	**NZ**	**CB**	**CG**	**CD**	**CE**	**NZ**
**C23**	0.01	0.01	0.02	0.01	0.01	0.01	0.01	0.01	0.01	0.00
**C24**	0.01	0.01	0.01	0.01	0.01	0.00	0.00	0.01	0.01	0.00
**C25**	0.01	0.01	0.01	0.01	0.00	0.00	0.00	0.01	0.01	0.00
**C26**	0.02	0.01	0.00	0.00	0.00	0.00	0.00	0.01	0.00	0.00
**C27**	0.03	0.01	0.01	0.00	0.00	0.01	0.01	0.01	0.01	0.00
**C28**	0.02	0.01	0.01	0.01	0.00	0.01	0.01	0.02	0.01	0.01

[a] Probabilities in orange represent those exceeding 0.03.

References

[1] Han, M. and Hansmann, U. H. E. (2011) Replica exchange molecular dynamics of the thermodynamics of fibril growth of Alzheimer's Aβ42 peptide. *J. Chem. Phys.* **135**, 065101.

[2] Denschlag, R., Lingenheil, M., and Tavan, P. (2009) Optimal temperature ladders in replica exchange simulations. *Chem. Phys. Lett.* **473**, 193-195.

[3] Delfing, B. M., Laracuente, X. E., Olson, A., Foreman, K. W., Paige, M., Kehn-Hall, K., Lockhart, C., and Klimov, D. K. (2023) Binding of Viral Nuclear Localization Signal Peptides to Importin-α Nuclear Transport Protein. *Biophys. J.* **122**, 3476-3488.

[4] Delfing, B. M., Laracuente, X. E., Jeffries, W., Luo, X., Olson, A., Foreman, K. W., Petruncio, G., Lee, K. H., Paige, M., Kehn-Hall, K., Lockhart, C., Klimov, D. K. (2024) Competitive Binding of Viral Nuclear Localization Signal Peptide and Inhibitor Ligands to Importin-alpha Nuclear Transport Protein. *J. Chem. Inf. Model.* **64**, 5262-5272.

[5] Delfing, B. M., Olson, A., Laracuente, X., Foreman, K. W., Paige, M., Kehn-Hall, K., Lockhart, C., and Klimov, D. K. (2023) Binding of Venezuelan Equine Encephalitis Virus Inhibitors to Importin-α Receptors Explored with All-Atom Replica Exchange Molecular Dynamics. *J. Phys. Chem.* B **127**, 3175–3186.

[6] Genheden, S and Ryde, U. (2015) The MM/PBSA and MM/GBSA methods to estimate ligand-binding affinities. *Expert Opin. Drug Discov*. **10**, 449-461.

[7] Roux, B. and Chipot, C. (2024) Editorial Guidelines for Computational Studies of Ligand Binding Using MM/PBSA and MM/GBSA Approximations Wisely. *J. Phys. Chem. B* **128**, 12027-12029.
