# Supplementary material for: Ligands binding diffusively to protein target act as inhibitors of protein-protein interactions
Source: PLoS Comput Biol. 2025 Sep 17;21(9):e1013495. doi: 10.1371/journal.pcbi.1013495 (PMC12456801; doi:10.1371/journal.pcbi.1013495)
Supplement: S2 Text — (PDF) [file pcbi.1013495.s002.pdf]

Supporting Information 2

Ligands binding diffusively to protein target act as inhibitors of protein-protein interactions

William Jeffries[1], Bryan M. Delfing[1], Xavier E. Laracuente[1], Xingyu Luo[1], Audrey Olson[1], Kenneth W. Foreman[2], Kyung Hyeon Lee[2,3], Greg Petruncio[2,3], Vito De Benedictis[2,3], Mikell Paige[2,3], Kylene Kehn-Hall[4,5], Christopher Lockhart[1], and Dmitri K. Klimov[1*]

[1]School of Systems Biology, George Mason University, Manassas, Virginia, United States of America
[2]Department of Chemistry and Biochemistry, George Mason University, Manassas, Virginia, United States of America
[3]Center for Molecular Engineering, George Mason University, Manassas, Virginia, United States of America
[4] Department of Biomedical Sciences and Pathobiology, Virginia-Maryland College of Veterinary Medicine, Virginia Polytechnic Institute and State University, Blacksburg, Virginia, United States of America
[5]Center for Emerging, Zoonotic, and Arthropod-borne Pathogens, Virginia Polytechnic Institute and State University, Blacksburg, Virginia, United States of America

*E-mail: dklimov@gmu.edu

AlphaScreen Inhibition Assay

The inhibitory assay of ImpA in the presence of compounds DP9 and DP9o was determined by AlphaScreen assay in a 384-well Perkin-Elmer white opaque-bottom plate. 1 μL of a 1:10 dilution of the acceptor beads, 1 μL of 2.5% bovine serum albumin (BSA), and 1 μL of a 1:10 dilution of the donor beads was prepared in a dark room. Assays were performed in a total reaction volume of 25 μL containing 5 μL of His-tagged ImpA [2.5 nM], 5 μL of FLAG-tagged NLS [60 nM], 5 μL of DP9 or DP9o in escalating concentrations [0-800 μM], and 7 μL of 1×PBS pH 7.2 buffer in the presence of 3 μL of donor and acceptor beads. [REF: doi: 10.1016/j.ab.2005.10.029] The plate was incubated at room temperature away from light for 30 minutes and subsequently read on a Tecan Spark 10 M Spectrometer with Alpha Assay protocol. Samples were prepared in 6 replicates and IC_{50} values were calculated by fitting to a four-parameter nonlinear regression curve in GraphPad Prism 10.

COMPOUND SYNTHESIS PROCEDURES

General: Special instructions regarding reagents are noted in the appropriate procedures where they are employed. "Drying an organic layer" refers to treatment with Na_2SO_4 or $MgSO_4$ after aqueous workup and then decanting. "Flame-drying" refers to heating glassware with a propane torch for one minute immediately prior to use. "Flushing/purging a reaction vessel" refers to delivering an inert gas using balloon+syringe+needle system through a septum for several minutes. A Biotage Initiator was used for microwave reactions. Reaction progress was monitored on SiliCycle F^{254} TLC plates. "TLCMS" refers to MS analysis of spots on a TLC plate. "Concentrated" refers to removal of volatile solvent using Biotage V10 Touch or Buchi rotary evaporator with water bath temperature between 40-50 °C. Products were purified using SiliCycle SiliaFlash F60 silica gel (40-63 μm, 230-400 mesh) with solvent mixtures specified in the corresponding experiment.

Instrumentation: APCI MS was performed on an Advion Expression CMS. TLCMS was performed on Advion Expression CMS Plate Express attachment, which allows for direct APCI MS analysis of spots on a TLC plate. ^{1}H and ^{13}C NMR spectra were obtained on a Bruker 400 MHz spectrometer equipped with a PA BBO probe and spectra were calibrated using TMS or the solvent residual peak. LCMS and UV-trace analysis was run on a SciEx QTRAP 4100.

Chemicals: Hippuric acid, aryl aldehydes, 1-(3-aminopropyl)imidazole, and acetic anhydride were purchased from commercial suppliers and used without further purification. Triethylamine and dichloromethane were purchased from commercial suppliers and stored over KOH or molecular sieves, respectively. Reagent alcohol grade was used for ethanol.

Overall Scheme: Synthesis of DP9 and DP9-*ortho*

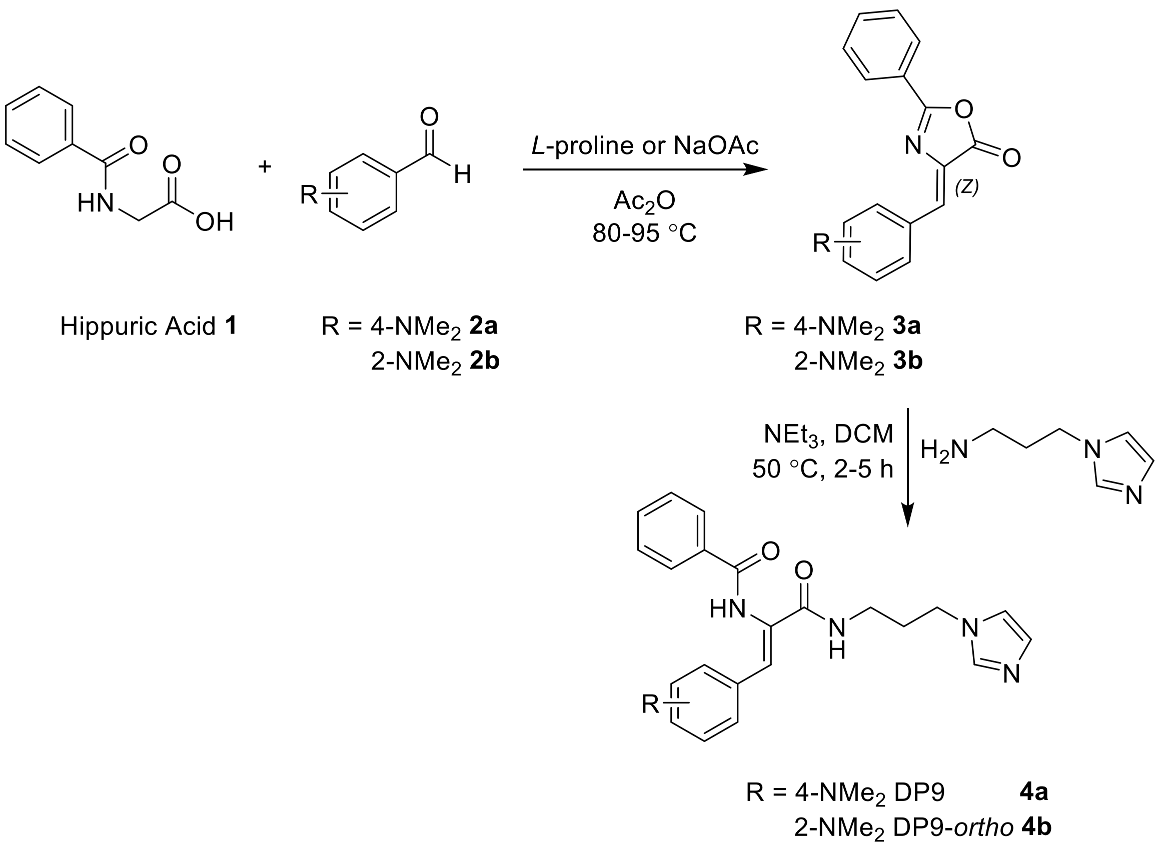

DP9 SYNTHESIS

Step 1: Knoevenagel condensation

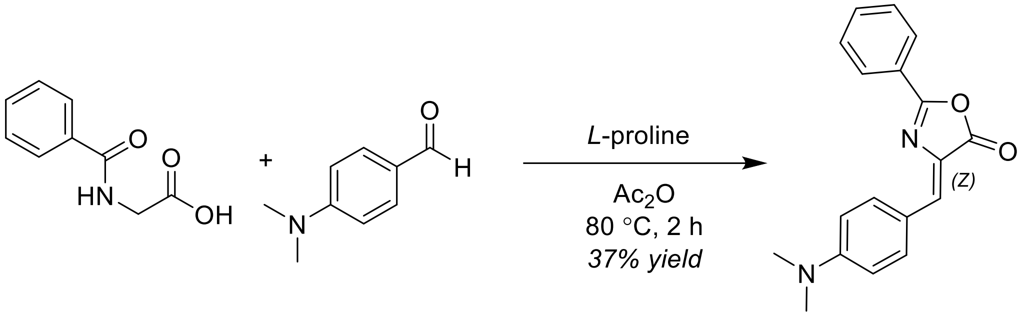

1 **2a** **3a**

Procedure: The procedure was adopted from Bhandari *et al.*[1] To a screw cap vial was added hippuric acid (**1**) (896 mg, 5 mmol, 1 equiv.), 4-dimethylaminobenzaldehyde (**2a**) (746 mg, 5 mmol, 1 equiv.), *L*-proline (57 mg, 0.5 mmol, 10 mol%), and acetic anhydride (2.8 mL, 30 mmol, 6 eq.). The reaction was then placed in an 80 °C oil bath and stirred for 2 h. Upon cooling to room temperature, the reaction product crystallized. To the reaction mixture was added boiling water (~30 mL), which was used to transfer the reaction suspension to a falcon tube. The reaction was centrifuged and the water was decanted. This process was repeated one more time. Boiling ethanol was added to the remaining red solid in the falcon tube and the homogenous solution was then transferred to a beaker for recrystallization. A red crystalline solid was vacuum dried to obtain 539 mg (37% yield).

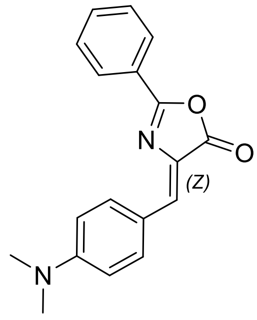

3a

^{1}H NMR (400 MHz, DMSO-d6): δ 3.08 (6H, s, -N(Me)$_2$), 6.83-6.85 (2H, d, ArH), 7.24 (1H, s, -CH=), 7.59-7.69 (3H, m, ArH), 8.06-8.08 (2H, d, ArH), 8.17-8.20 (2H, d, ArH) ppm.

^{13}C NMR (125 MHz, DMSO-d6): δ: 41.1, 111.95, 113.59, 124.92, 126.01, 126.67, 127.16, 127.49, 129.17, 130.51, 150.64, 157.20, 160.24.

Step 2: Lactone Amidation

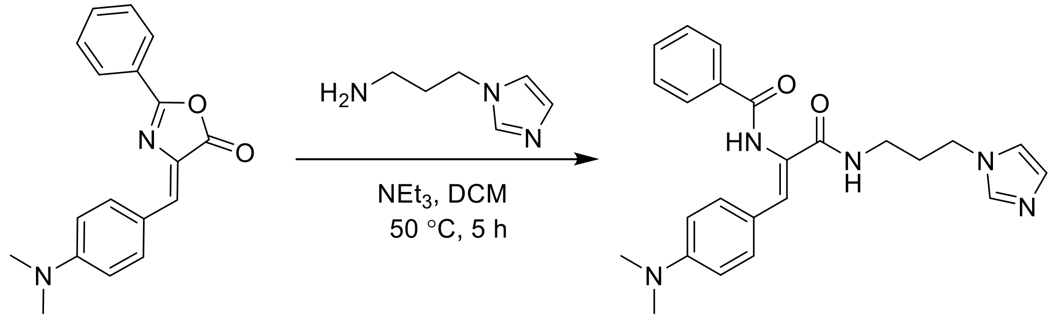

3a **4a**
DP9

Procedure: Azalactone **3a** (620 mg, 2.1 mmol, 1 equiv.) was dissolved in 15 mL of DCM and transferred to a microwave vial. 1-(3-aminopropyl)imidazole (385 µL, 3.08 mmol, 1.5 equiv.) was then added followed by triethylamine (432 µL, 3.08 mmol, 1.5 equiv.). The reaction was heated to 50 °C in the microwave for 5 h. TLC indicated reaction completion. Water was added to the reaction vial and the biphasic mixture was stirred vigorously at room temp. for several minutes. The layers were separated and the organic layer was washed with another portion of water and then with brine. The organic layer was dried, concentrated, and the crude was loaded onto a silica gel column and eluted with 5:95 MeOH-DCM and then 1:5:94 NH_4OH-MeOH-DCM. The obtained product was isolated as 730 mg of a yellow solid (82% yield). The stereochemistry of the double bond was assumed to be *Z* based on verification of a related analogue's stereochemistry by 2D NOESY.

TLC system: 1:1 EtOAc-Hex (*From left to right: sm = starting material* **3a***, rxn = reaction, co = cospot*)

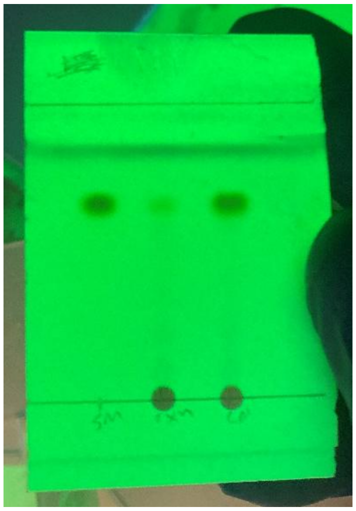

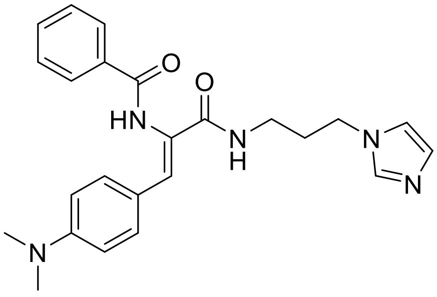

4a
DP9

^{1}H NMR (400 MHz, DMSO-d6) δ 1.86-1.89 (tt, 2H, CH_2), 2.92 (s, 6H, -$N(CH_3)_2$), 3.11-3.15 (td, 2H, CH_2), 3.96-4.00 (t, 2H, CH_2), 6.64-6.66 (d, 2H, ArH), 6.87 (s, 1H, =CH), 7.16-7.18 (d, 2H, Imidazole-H), 7.43-7.45 (d, 2H, ArH), 7.53-7.62 (m, 4H, Imidazole-H, ArH), 8.00-8.07 (dt, 3H, ArH, -NH), 9.81 (s, 1H, -NH) ppm.

^{13}C NMR (125 MHz, DMSO-d6) δ 25.95, 36.4, 40.30, 40.30, 49.56, 117.98, 118.44, 111.88, 111.88, 127.72, 127.72, 128.63, 128.63, 128.92, 128.8, 129.10, 129.10, 129.16, 131.89, 133.53, 137.31, 151.43, 158.82, 165.6.

Purity (LC-UV trace): 99%

APCI-MS $[M+1]^+$: 417.9

DP9-*ortho* SYNTHESIS

Steps 1-2: Telescoped Knoevenagel condensation & Lactone Amidation

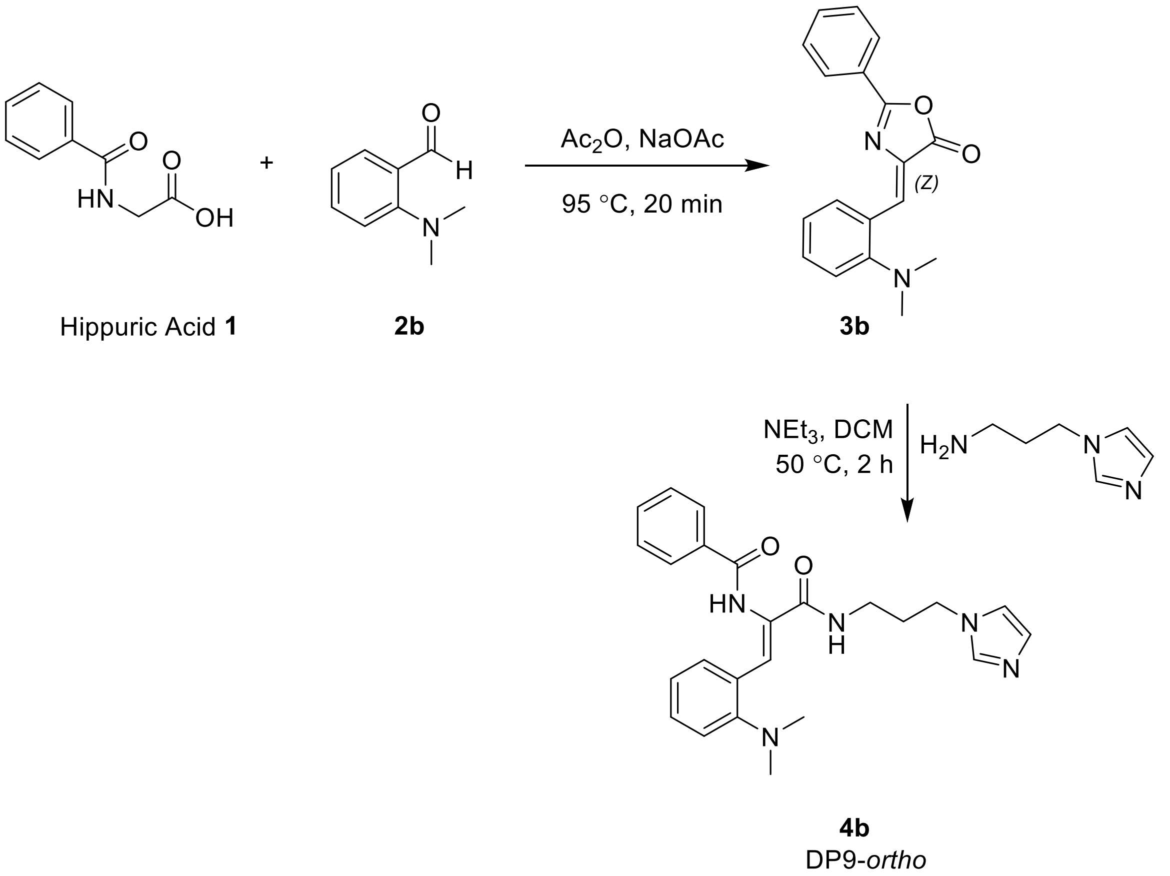

Procedure: To a microwave vial was added hippuric acid (**1**) (82 mg, 0.47 mmol, 1 equiv.), 2-dimethylaminobenzaldehyde (**2b**) (70 mg, 0.47 mmol, 1 equiv.), NaOAc (77 mg, 0.94 mmol, 2 equiv.) and acetic anhydride (~3 mL). The reaction was then heated to 95 °C in the microwave for 20 min. Upon cooling to room temperature, the reaction product crystallized. The crude was recrystallized from ethanol and the product was immediately subjected to the next reaction without characterization. Intermediate azalactone **3b** (50 mg, 0.17 mmol, 1 equiv.) was dissolved in 3 mL of DCM and transferred to a microwave vial. 1-(3-aminopropyl)imidazole (31 µL, 0.26 mmol, 1.5 equiv.) was then added followed by triethylamine (36 µL, 0.26 mmol, 1.5 equiv.). The reaction was heated to 50 °C in the microwave for 2 h. TLC analysis indicated appearance of the product. The reaction was washed with water (3×3 mL). The organic layer was dried, concentrated, and the crude product was loaded onto a silica gel column and eluted with 5:95 MeOH-DCM and then 1:5:94 NH_4OH-MeOH-DCM. The product was obtained as 30 mg (15% yield over two steps, 42% yield second step). The stereochemistry of the double bond was assumed to be *Z* based on verification of a related analogue's stereochemistry by 2D NOESY.

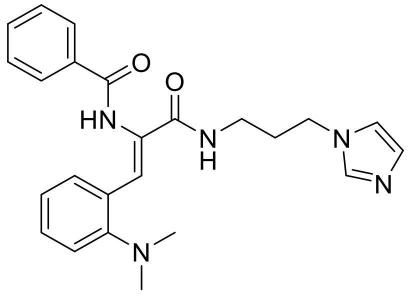

4b
DP9-*ortho*

^{1}H NMR (400 MHz, DMSO-d6) δ 10.01 (broad s, 1H), 8.21-8.18 (t, 1H, *J* = 5.5 Hz), 7.92-7.90 (d, 2H, *J* = 7.4 Hz), 7.65 (s, 1H), 7.59-7.55 (t, 1H, *J* = 7.8 Hz), 7.52-7.48 (m, 2H), 7.44-7.42 (d, 1H, *J* = 7.6 Hz), 7.23-7.20 (m, 2H), 7.15 (s, 1H), 7.07-7.05 (d, 1H, *J* = 8.0 Hz), 6.90-6.89 (m, 2H), 4.03-4.00 (t, 2H, *J* = 6.9 Hz), 3.18-3.13 (q, 2H, *J* = 6.1 Hz), 2.74 (s, 6H), 1.92-1.89 (t, 2H, *J* = 6.6 Hz)

^{13}C NMR (125 MHz, DMSO-d6) δ 165.55, 165.33, 151.98, 137.30, 133.93, 131.63, 129.91, 129.25, 129.00, 128.30, 127.88, 127.74, 127.48, 125.50, 121.30, 119.36, 117.71, 44.10, 43.48, 36.17, 30.85

APCI-MS $[M+1]^+$: 418.0

SPECTRA

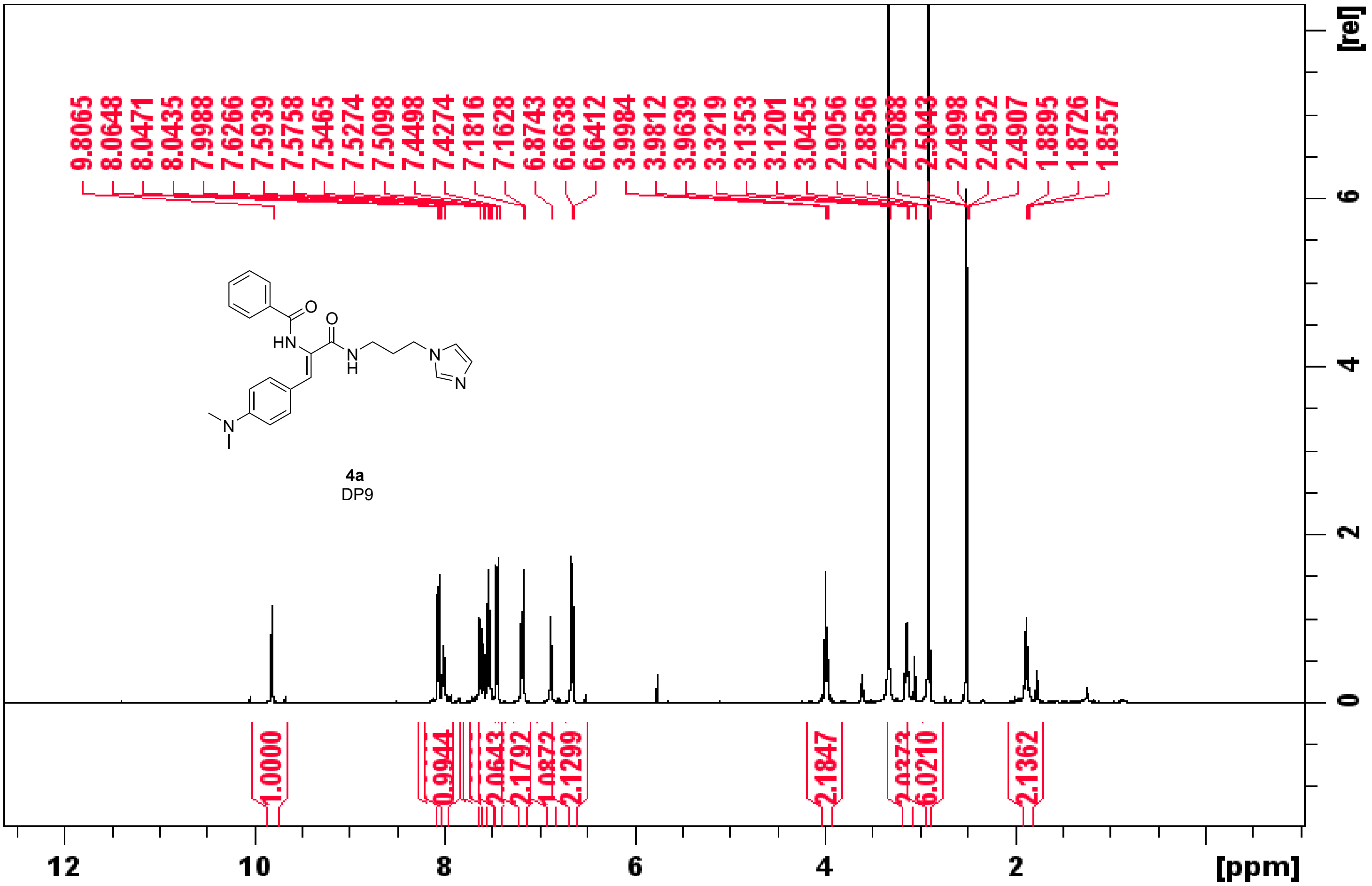

4a
DP9

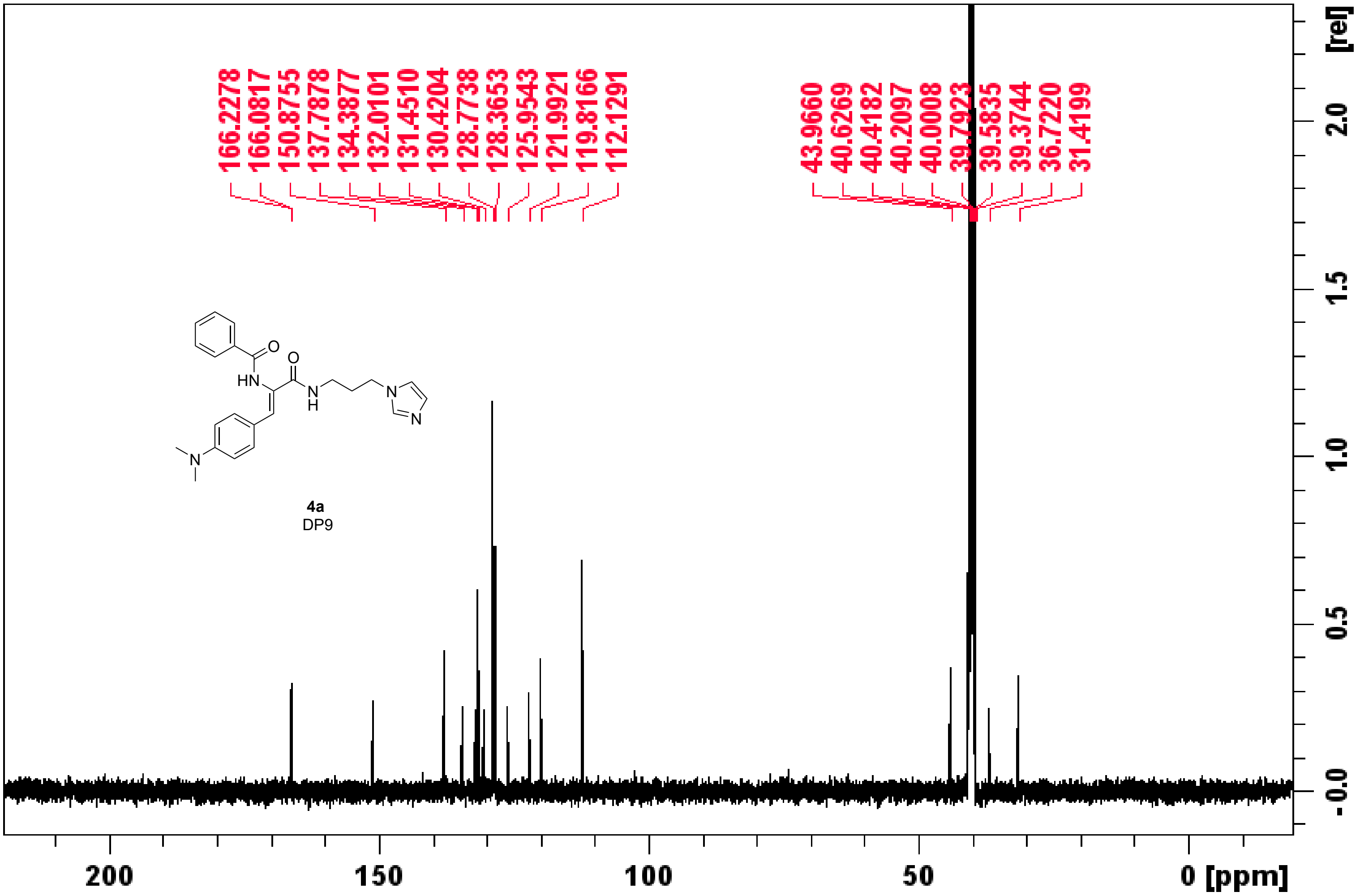
166.2278
166.0817
150.8755
137.7878
134.3877
132.0101
131.4510
130.4204
128.7738
128.3653
125.9543
121.9921
119.8166
112.1291
43.9660
40.6269
40.4182
40.2097
40.0008
39.7923
39.5835
39.3744
36.7220
31.4199
HN
O
O
N
H
N
N
N
4a
DP9
[rel]
2.0
1.5
1.0
0.5
-0.0
200
150
100
50
0 [ppm]

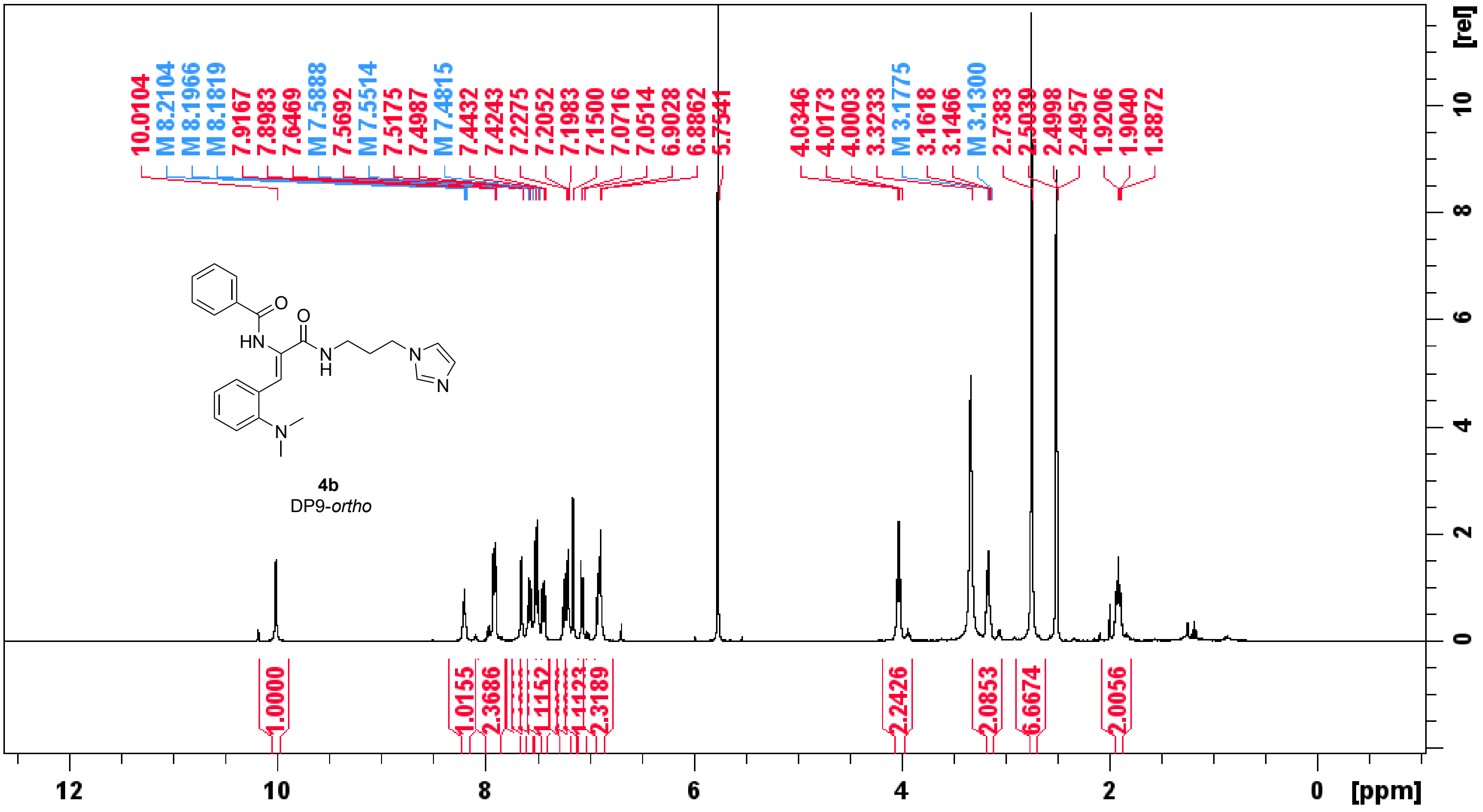
10.0104
M 8.2104
M 8.1966
M 8.1819
7.9167
7.8983
7.6469
M 7.5888
7.5692
M 7.5514
7.5175
7.4987
M 7.4815
7.4432
7.4243
7.2275
7.2052
7.1983
7.1500
7.0716
7.0514
6.9028
6.8862
5.7541
4.0346
4.0173
4.0003
3.3233
M 3.1775
3.1618
3.1466
M 3.1300
2.7383
2.5039
2.4998
2.4957
1.9206
1.9040
1.8872
1.0000
1.0155
2.3686
1.1152
1.1123
2.3189
2.2426
2.0853
6.6674
2.0056
4b
DP9-*ortho*
[ppm]
[rel]

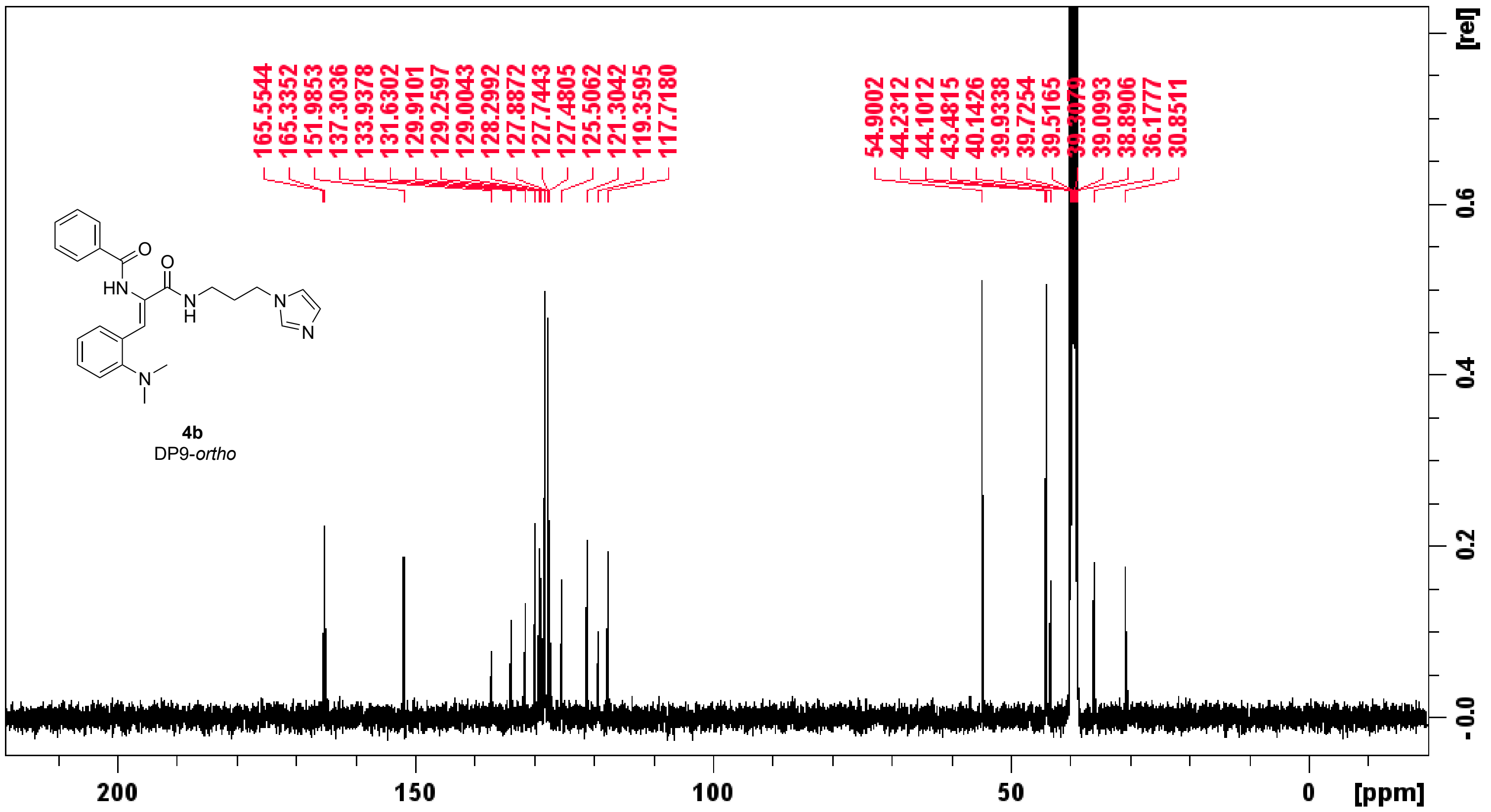

165.5544
165.3352
151.9853
137.3036
133.9378
131.6302
129.9101
129.2597
129.0043
128.2992
127.8872
127.7443
127.4805
125.5062
121.3042
119.3595
117.7180
54.9002
44.2312
44.1012
43.4815
40.1426
39.9338
39.7254
39.5165
39.3079
39.0993
38.8906
36.1777
30.8511
HN
O
O
N
H
N
N
N
4b
DP9-ortho
200
150
100
50
0
[ppm]
[rel]
0.6
0.4
0.2
- 0.0
